# PM_2.5_ Is Insufficient to Explain Personal PAH Exposure

**DOI:** 10.1029/2023GH000937

**Published:** 2024-02-10

**Authors:** Lisa M. Bramer, Holly M. Dixon, Diana Rohlman, Richard P. Scott, Rachel L. Miller, Laurel Kincl, Julie B. Herbstman, Katrina M. Waters, Kim A. Anderson

**Affiliations:** ^1^ Biological Sciences Division Pacific Northwest National Laboratory Richland WA USA; ^2^ Department of Environmental and Molecular Toxicology Food Safety and Environmental Stewardship Program Oregon State University Corvallis OR USA; ^3^ College of Health Oregon State University Corvallis OR USA; ^4^ Division of Clinical Immunology Icahn School of Medicine at Mount Sinai New York City NY USA; ^5^ Department of Environmental Health Sciences Columbia Center for Children's Environmental Health Mailman School of Public Health Columbia University New York City NY USA

**Keywords:** silicone wristband, AQI, wildfire smoke, PAH, exposome, mixtures

## Abstract

To understand how chemical exposure can impact health, researchers need tools that capture the complexities of personal chemical exposure. In practice, fine particulate matter (PM_2.5_) air quality index (AQI) data from outdoor stationary monitors and Hazard Mapping System (HMS) smoke density data from satellites are often used as proxies for personal chemical exposure, but do not capture total chemical exposure. Silicone wristbands can quantify more individualized exposure data than stationary air monitors or smoke satellites. However, it is not understood how these proxy measurements compare to chemical data measured from wristbands. In this study, participants wore daily wristbands, carried a phone that recorded locations, and answered daily questionnaires for a 7‐day period in multiple seasons. We gathered publicly available daily PM_2.5_ AQI data and HMS data. We analyzed wristbands for 94 organic chemicals, including 53 polycyclic aromatic hydrocarbons. Wristband chemical detections and concentrations, behavioral variables (e.g., time spent indoors), and environmental conditions (e.g., PM_2.5_ AQI) significantly differed between seasons. Machine learning models were fit to predict personal chemical exposure using PM_2.5_ AQI only, HMS only, and a multivariate feature set including PM_2.5_ AQI, HMS, and other environmental and behavioral information. On average, the multivariate models increased predictive accuracy by approximately 70% compared to either the AQI model or the HMS model for all chemicals modeled. This study provides evidence that PM_2.5_ AQI data alone or HMS data alone is insufficient to explain personal chemical exposures. Our results identify additional key predictors of personal chemical exposure.

## Key Points

- Explaining personal chemical exposures required more than fine particulate matter air quality index (AQI) or hazard mapping system data
- Models with variables in addition to fine particulate matter AQI increased predictive accuracy of exposure
- Heavy wildfire smoke was measured during the study

## 1 Introduction

To understand how chemical exposure can impact health, especially during periods of poor air quality and wildfires, researchers need tools that capture the complexities of personal chemical exposure. Chemical‐related health outcomes can be a result of exposure to chemicals adsorbed to the surface of particulate matter (PM), the PM itself, and chemicals present in the gas‐phase that make it into the body. It is challenging to assess all of these different types of possible chemical exposures so, in practice, environmental epidemiology studies rely on a portion of total chemical exposure when linking chemical exposure to health outcomes. For instance, the U.S. Environmental Protection Agency (EPA) states that PM_2.5_ is typically the pollutant that is monitored and used to estimate public health effects from wildfire smoke because other air pollutants (e.g., polycyclic aromatic hydrocarbons (PAHs)) are difficult to measure during a smoke incident (EPA, 2021).

Many environmental epidemiology studies rely on publicly available data sets containing limited chemical measures as proxies for total personal chemical exposure (J. C. Liu et al., 2015). Example measures include PM data from stationary air monitors and wildfire smoke density data from satellites (Gan et al., 2017). Numerous studies have analyzed the relationship between wildfire smoke and health outcomes using PM stationary air monitoring data, HMS data, or a combination of both as proxies for personal chemical exposure (Abdo et al., 2019; Aguilera, Corringham, Gershunov, Leibel, & Benmarhnia, 2021; Aguilera, Corringham, Gershunov, & Benmarhnia, 2021; Cleland et al., 2022; Dhingra et al., 2023; Heft‐Neal et al., 2022; Jones et al., 2020; Lipner et al., 2019; Magzamen et al., 2021; Mirabelli et al., 2022; O’Dell et al., 2022; Sorensen et al., 2021; Stowell et al., 2019; Wettstein et al., 2018). For example, a recent wildfire epidemiology study compared mental health symptoms with HMS smoke categories and stated that the HMS estimates are “a proxy for exposure to wildfire smoke” (Mirabelli et al., 2022). While frequently used, it is not understood how well these exposure measures represent the complexity of personal chemical exposure, which is necessary to draw valid conclusions and make recommendations regarding human health.

PM_2.5_ is the form of PM regularly used as a proxy for personal exposure (J. C. Liu et al., 2015). PM_2.5_ is the fraction of particulate matter with diameters of 2.5 μm and smaller that settles deep in the lungs (EPA, 2022b). The U.S. EPA calculates an air quality index (AQI) for PM_2.5_, an index that is relative to the short‐term national ambient air quality standard for protection of public health (AirNow, 2022). PM_2.5_ concentrations are also widely measured and consistently high during wildfire smoke events (Naeher et al., 2007; Sapkota et al., 2005; Yao et al., 2013). Wildfire smoke density information is captured by the U.S. National Oceanic and Atmospheric Administration's (NOAA's) HMS (Ruminski et al., 2008), and is also used as a proxy for chemical exposure in recent research (J. C. Liu et al., 2015; Mirabelli et al., 2022), although used less often as a proxy in comparison to PM_2.5_.

However, a key limitation is that widely available PM_2.5_ data does not account for chemicals adsorbed to the PM or gas‐phase chemicals. Recent research has characterized and highlighted the importance of gas‐phase chemical exposures during wildfires (Ghetu et al., 2022; Messier et al., 2019). Wildfire smoke in particular is a complex chemical mixture and different wildfire fuel sources can contribute to different chemical mixtures (Rager et al., 2021; Samburova et al., 2016; Urbanski et al., 2008). PAHs are a chemical class found in wildfire smoke (Rager et al., 2021), which have been associated with a wide range of health effects including cancer (ATSDR, 2005; Moorthy et al., 2015). PAHs are present in both the gas‐phase and bound to particulate matter (Ghetu et al., 2022; Maharaj Kumari & Lakhani, 2018). In addition, researchers have found that gas‐phase organic chemicals are regularly an order of magnitude larger indoors than outdoors (Goldstein et al., 2021). Individual behaviors, such as time spent indoors or in transit, may also influence personal chemical exposure, which are not captured with geographically spread out PM_2.5_ outdoor stationary monitors.

Passive sampling silicone wristbands are a tool that can be used to quantify more individualized chemical exposure data than stationary air monitors or smoke satellites for a wide variety of volatile and semi‐volatile organic chemicals (VOCs and SVOCs), including PAHs. Wristbands are easy for participants to receive, wear, and return to researchers and wristband studies report high levels of participant compliance (Dixon et al., 2022). Thousands of study participants have worn wristbands since the first wristband study in 2014 (Samon et al., 2022). Wristbands integrate exposure information from inhalation, dermal, and dermal excretion exposure routes when worn directly on the wrist (Samon et al., 2022). Studies show that wristbands provide similar exposure assessment information as biological metabolites (Dixon et al., 2018, 2022; Hammel et al., 2016, 2018, 2020; Hoffman et al., 2021; Levasseur et al., 2021; Quintana et al., 2019; Xie et al., 2021). Given their ease‐of‐use and alignment with the portion of chemicals in the body following exposure, wristbands are also being used in studies to link chemical exposure with health outcomes (Samon et al., 2022).

It is not understood how PM_2.5_ data from stationary air monitors and wildfire smoke density data from satellites align with personal chemical exposure data from silicone wristbands; we present the first study to evaluate this. We had participants wear silicone wristbands, carry a phone that recorded global positioning system (GPS) locations, and answer daily questions. We also gathered daily PM_2.5_ AQI and HMS data from the U.S. EPA and NOAA. We hypothesized that predictive models for personal organic chemical exposure would be significantly improved by expanding beyond stationary PM_2.5_ AQI or satellite HMS data to also include environmental and behavioral information. The results of this study provide evidence to inform additional key predictors of personal chemical exposure.

To the authors' knowledge, this is the largest data set used to investigate the predictive value of both environmental and behavioral variables on personal chemical exposure, including over 40,000 chemical concentrations from wristbands. This is also the first wristband study to characterize non‐occupational wildfire smoke exposure. This study is further strengthened by the inclusion of repeated daily measures from participants across different seasons, chemical exposure data during wildfire events, the use of interpretable multivariate statistical learning models, and over 50,000 participant‐specific GPS locations.

## 2 Materials and Methods

### 2.1 Study Cohort and Design

This study is part of a larger research project; other complementary studies focus on other types of data such as participant's lung function (Evoy et al., 2022; Rohlman et al., 2019). To participate in the study, people had to meet the following criteria: (a) be age 18 or older, (b) have a current asthma diagnosis, (c) have mild to moderate asthma as assessed by asthma severity questions, (d) be a current non‐smoker, and (e) live within a 20‐mile radius of Eugene, Oregon, U.S. Of the 47 individuals recruited, 38 enrolled in the study. All participants gave informed verbal and written consent. This study was approved by Oregon State University's Institutional Review Board (#IRB‐2020‐0899).

We asked each participant to participate in both summer and winter to examine seasonal differences in chemical exposures. Summer dates ranged from 7 August to 4 October in 2017 and 2018. Winter dates ranged from 6 February to 11 April in 2018. Thirty‐five people completed at least one season of the study (92%). Of these 35 people, 29 completed the study in both seasons (76%). Six people only completed one season, three in the summer and three in the winter.

A majority of study participants identified as female (80%) and white (86%; Table 1). Age ranged from 21 to 74, with a mean age of 48 (Table 1). Table A1 in Supporting Information S1 includes participant demographics by season.

**Table 1:** Study Participant Demographics

Continuous characteristic	Range	Mean ± SD
Age (years)	21–74	48 ± 15

Categorical characteristics	Number of people	Percent out of 35
Gender		
Female	28	80.0
Male	7	20.0
Education Level		
High School	2	5.7
Some College	6	17.1
Associate's Degree	5	14.3
Bachelor's Degree	12	34.3
Master's Degree	8	22.9
Doctoral Degree	1	2.9
Prefer Not to Answer	1	2.9
Household Income		
Less than $39,999	14	40.0
$40,000 to $79,999	11	31.4
More than $80,000	8	22.9
Prefer Not to Answer	2	5.7
Race		
American Indian/Alaska Native	1	2.9
Black/African American	2	5.7
More than One Race	1	2.9
White	30	85.7
Unknown/Other	1	2.9
Ethnicity		
Hispanic or Latino	3	8.6
Not Hispanic or Latino	26	74.3
Prefer Not to Answer	6	17.1

This study uses the “ELF tool” that was initially developed in response to community concerns (Rohlman et al., 2015) and has been previously described (Evoy et al., 2022; Rohlman et al., 2019). This study also included repeated daily measures; participants used the ELF tool for a 7‐day period within a season. Participants kept a study‐provided Android cell phone with them throughout the study period, which had the ELF Tracker Application (App) on it (Rohlman et al., 2015). The App recorded and transmitted continuous GPS data and answers from short questionnaires to our secure data management system at Pacific Northwest National Laboratory (PNNL). Individuals put on a new wristband each day, wearing a total of seven different wristbands each season. The wristband, GPS, and questionnaire components of the ELF tool are described in additional detail below.

We included 364 wristbands worn by 35 study participants in the final data set used for modeling. Figure A1 in Supporting Information S1 includes a flowchart of how we paired the data sources together and includes summary values like data set size for each source (i.e., wristbands, GPS, questionnaire, PM_2.5_ AQI, and HMS).

### 2.2 Wristband Methodology

#### 2.2.1 Preparation and Deployment

We prepared silicone wristbands (24hourwristbands.com, Houston, TX, USA) as previously described (Anderson et al., 2017). We rinsed wristbands with deionized water and used a vacuum oven (300°C for 12 hr at 0.1 Torr; Blue M vacuum oven, no. POM18VC; Welch^®^ DuoSeal pump, no. 1405, Mt. Prospect, IL, U.S.) to remove manufacturing process‐related chemicals in the silicone polymer. We collected and analyzed quality control (QC) samples from each group of prepared wristbands to ensure all wristbands met our data quality objectives, which are described in detail in Dixon et al. (2019). Additional information on laboratory materials, deployment, and QC is in Supporting Information S1.

#### 2.2.2 Inclusion and Exclusion Criteria

Once participants turned in their ELF kits, we evaluated if each wristband met our inclusion criteria to eliminate those that may not be representative of daily chemical exposure. We only analyzed wristbands that (a) were worn within ±8 hr of the 24‐hr period, (b) had recorded date and time information for when the wristband was put on and taken off, and (c) were in airtight polytetrafluorethylene bags upon receipt. In addition, we did not analyze wristbands worn beyond the 7‐day study period. We required wristbands to be put on between 12:01 a.m. and 11:59 a.m. Overall, we excluded 22 wristbands (5% of all wristbands collected) that did not meet the inclusion criteria.

#### 2.2.3 Cleaning and Extraction

We cleaned the 426 wristbands that met our inclusion criteria and extracted the chemicals from the silicone as previously described (Dixon et al., 2022). Particles on the wristband surface do not reflect the chemical fraction available to be absorbed by the body, and we removed particles with two rinses of 18 MΩ*cm water and one rinse of isopropanol (Anderson et al., 2017; Dixon et al., 2022). We extracted chemicals by adding two separate 50 mL volumes of ethyl acetate at room temperature to the wristbands. We quantitatively concentrated the ethyl acetate to one mL using TurboVap^®^ 500 closed cell evaporators and a TurboVap^®^ LV evaporator workstation (Biotage LLC, Charlotte, NC, USA).

#### 2.2.4 Chemical Analysis

We quantitatively analyzed all wristband extracts for 94 organic chemicals using an Agilent (Santa Clara, CA, U.S.) 7890A gas chromatograph (GC) interfaced with an Agilent 5975C mass spectrometer (MS) detector as well as an Agilent 6890N GC interfaced with an Agilent 5975B MS. We used an Agilent DB‐5MS GC column (30 m × 0.25 mm), and each chemical in the method was calibrated with a curve of at least five points (correlations ≥0.99). The method includes 94 VOCs and SVOCs including PAHs, OPAHs, tri‐r‐phosphates, and alkanes. All target analytes, molecular weights, limits of detection (LODs), and limits of quantitation (LOQs) are in Table A2 of Supporting Information S1.

In a few cases, we were not able to detect certain deuterated chemical surrogates in a minority of wristband extracts due to matrix interference. As a result, we were not able to quantify the target chemical related to the undetected chemical surrogate in these instances. Table A2 in Supporting Information S1 includes the number of wristbands for each target chemical that did not have matrix interference. Compounds in sweat or personal care products are potential reasons for matrix interference.

### 2.3 ELF Tracker App

#### 2.3.1 GPS

To pair the GPS data with the wristband data, we excluded 14 wristbands where the study participant reported in their daily questionnaire that (a) they did not carry the phone on their person or (b) the phone was not left in their general location (i.e., home or office) while wearing their wristband. We also filtered out one wristband that did not correspond to any GPS time points, which may have been due to the phone being off.

Quality control was performed on each participant's GPS location data and is described in Appendix A (Text A2 and Figure A2 in Supporting Information S1). For each wristband sampling period (about 24 hr), we matched any GPS observations that occurred between the wristband on and off times to the corresponding sampling period.

#### 2.3.2 Questionnaire

For this study, we analyzed answers from two different questions in the evening questionnaire: (a) did you carry the phone on your person today; (b) how much time did you spend indoors today (0, <1, 1–8, 9–12, or 12–24 hr)? If participants answered no to the first question, they were then asked to select a reason why they did not carry the phone on them: forgot, inconvenient, left in my general location (home, office, etc.), device malfunction, or other. We used this information to filter the GPS data.

To compare questionnaire data between days and to chemical exposure, we required evening questionnaires to be recorded between 4 p.m. and 4 a.m. A total of 47 questionnaires were not filled out during the required time period or were not filled out. Evening questionnaire data was then merged with wristband and GPS data, which resulted in data for 364 wristband sampling periods.

To aid in investigating questionnaire trends, we downloaded NOAA daily maximum and averages, dry bulb globe temperature, and relative humidity from the monitor in Eugene (station ID = WBAN:24221; data downloaded 3‐5‐2023; NOAA, 2023a). We computed the heat index based on maximum and mean dry bulb globe temperature (Steadman, 1979).

### 2.4 PM_2.5_ AQI

We downloaded daily PM_2.5_ AQI data for Oregon counties in 2017 and 2018 from the U.S. EPA (EPA, 2022a). There are other air pollutants that AQI can be calculated for; however, we analyzed PM_2.5_ AQI data here because (a) the PM_2.5_ monitoring network in Oregon is more extensive than other air pollutants, leading to a large data set that captured meaningful variability, (b) PM_2.5_ is often used a proxy for personal chemical exposure, and (c) AQI values are widely recognized and regularly used to decide public health messages. A detailed explanation of how the EPA calculates AQI is in the EPA's Technical Assistance Document for the Reporting of Daily Air Quality (EPA, 2018).

Forty‐nine AQI monitors in Oregon collected PM_2.5_ AQI data during our study period in 2017 and 2018 (Figure A3 in Supporting Information S1). PM_2.5_ AQI data is available for every day of the study. However, PM_2.5_ AQI data is not available from all monitors for every day; in any given month, daily data is available from 40 to 47 monitors. The PM_2.5_ AQI data files include latitude and longitude location. In some cases, air monitors recorded multiple PM_2.5_ AQI data points in a given day; in these instances (6.5% of all daily observations), we calculated the mean of the PM_2.5_ AQI data for each day.

We derived daily PM_2.5_ AQI values for each wristband sampling period (*n* = 364). Because people move around and can be in close proximity to multiple AQI monitors in one day, we calculated time weighted values. For each GPS location observed, we determined the closest AQI monitor to an individual. We then calculated the length of time that an individual was closest to each AQI monitor. The daily PM_2.5_ AQI value for each sampling period was then calculated as a weighted average of all AQI monitors' measurements, where we used weights that were the proportion of the sampling period that the participant was closest to each respective monitor.

The median distance from a participant's GPS location to the nearest PM_2.5_ AQI monitor was 2.5 miles (range from 0.02 to 49 miles). Eighty‐five percent of distances to the nearest PM_2.5_ AQI monitor were less than 10 miles.

### 2.5 HMS

We downloaded smoke information for 2017 and 2018 from the NOAA HMS database, which includes coordinates of smoke polygons and smoke plume density for each day (NOAA, 2023b). Smoke analysis is based on visual classification of smoke plumes using Advanced Baseline Imager true‐color color imagery available during sunlight. NOAA characterizes observed smoke into three smoke density categories (light, medium, and heavy) based on smoke opacity in the satellite imagery. While HMS data does cover large geographic areas, cloud cover can greatly interfere with the smoke satellites also cannot collect data at night, cannot identify the height of smoke plumes, and may not detect dilute smoke (Brey et al., 2018; O’Dell et al., 2022; Ruminski et al., 2008).

Spatial smoke density data were available for every day wristbands were worn in the study. Because study participants regularly changed locations, sometimes in and out of areas with different smoke density categories, we calculated the proportion of time each wristband was worn in none, light, medium, and heavy smoke density categories.

An example smoke density map from 15 September 2017, is shown in Figure A4 of Supporting Information S1. This map was created using ArcMap version 10.8.1. Figure A4 in Supporting Information S1 includes the ESRI Streets basemap, smoke density shapefile, and an Oregon state boundary shapefile. Polygons were overlaid on the map and altered by color and transparency to present the best view. The map uses the WGS 1984 projection.

### 2.6 Data Analysis

GPS location and questionnaire data were directly transmitted to secure servers at PNNL from the ELF Tracker App. The data acquisition and management system were previously published (Rohlman et al., 2019).

#### 2.6.1 Variable Construction for Modeling

We converted chemical concentrations to moles per gram wristband and log transformed the values (log_2_ pmol/g wristband). We set the concentration value to NA if there was matrix interference for a given chemical, which occurred for 1.3% of chemical concentration values. Additionally, for each chemical, we constructed low, medium, and high tertile categories based on concentrations. If the percentage of observations below the LOD was less than or equal to 33.33%, empirical percentiles of 33.33 and 66.67 were used to define thresholds for the tertiles. If the percentage of observations below the LOD was greater than 33%, all X% of observations below LOD were assigned to the low category and the remaining observations were divided into medium and high based on the [X + (100 − X)/2]^th^ percentile.

We calculated a single variable, HMS Index, to capture smoke density exposure. HMS Index was calculated as (0 × None + 1 × Light + 2 × Medium + 3 × Heavy)/3, where None, Light, Medium, and Heavy were the proportion of time spent in each density category. The HMS Index equals 0 when an individual spent no time in smoke and 1 when an individual spent all of the wristband sampling period in heavy smoke density.

Additionally, for each wristband sampling period, we calculated the total distance an individual traveled by summing the distances between consecutive observed GPS locations. We then created a categorical distance traveled variable by dividing the data into four category levels using the 25th, 50th, and 75th percentiles which were 4, 15, and 35 miles, respectively.

#### 2.6.2 Univariate Statistical Analyses

We conducted all statistical analyses using the statistical software R, version 4.1.2 (R Development Core Team, 2023).

We tested for differences in chemical detection by season, differences in mean concentration of observations above the LOD by season, and differences in chemical detection based on PM_2.5_ AQI or HMS Index. We required that a chemical was detected above LOD for at least three wristbands in one season and that the statistical model converged to conduct a test for differences in mean detection probability by season. A total of 52 of the 94 chemicals met this requirement. A mixed effects generalized linear model with a conditional binomial distribution (Dunn & Smyth, 2018) was fit to each chemical with season, PM_2.5_ AQI, or HMS as a fixed effect, for each respective test, and participant as a random effect.

We tested for difference in mean log_2_ concentration between seasons amongst observations that were above the LOD. We required that at least two seasons each had a minimum of two values above the LOD to conduct this test. A total of 53 of the 94 chemicals met this requirement. A mixed effects linear model with a conditional normal distribution (Dunn & Smyth, 2018) was fit to each chemical with season as a fixed effect and participant as a random effect. For both models, pairwise post‐hoc comparisons of summer 2017 (S17) versus summer 2018 (S18), S17 versus winter 2018 (W18), and S17 versus W18 were conducted to test for difference in detection probability between two seasons.

We used the *lme4* v.1.1‐40 package (Bates et al., 2015) to fit all mixed effects models, and we applied a Tukey correction for multiple comparisons (Hothorn et al., 2008) to compute adjusted p‐values.

We tested for differences in mean distance traveled between seasons by fitting an analysis of variance model (Faraway, 2002) to the log‐transformed distances and conducting all pairwise post‐hoc seasonal comparisons. *P*‐values for comparisons were corrected using a Tukey multiple test correction (Hothorn et al., 2008). We tested for differences in mean weighted AQI between seasons using Welch's *t*‐test (Welch, 1947), accounting for unequal variances, for each pairwise comparison and adjusted for multiple comparisons using a Holm adjustment (Hothorn et al., 2008). Because HMS Index is constrained between 0 and 1, we tested for the median difference in HMS Index observations for two seasons using a Wilcoxon non‐parametric test (Conover, 1999) and adjusted for multiple comparisons using a Holm adjustment (Hothorn et al., 2008).

#### 2.6.3 Correlative Statistical Analyses

To evaluate the shared information between two variables, we calculated a Spearman correlation (Myers & Well, 2003). We computed this correlation between AQI and HMS Index. Additionally, for each chemical where 50% or less of observations were below the LOD, we computed correlations between AQI and concentrations for each chemical and HMS and concentrations for each chemical.

#### 2.6.4 Machine Learning Modeling

For each chemical, we constructed several machine learning models with the end goal of predicting the exposure category (low, medium, or high) of wristbands. Specifically, we used classification tree machine learning models which provide the advantage of accounting for multiple predictor variables while providing interpretable results indicating which predictors are most important to the model. Because machine learning models are sensitive to class imbalance (i.e., one category with many more members; Abd Elrahman & Abraham, 2013), we limited our analysis to chemicals with no more than 50% of observations below LOD to ensure no more than a 2:1 ratio of the category size for the largest and smallest categories. A total of 25 chemicals met these requirements and are listed in Table A3 of Supporting Information S1.

We considered two baseline models that were fit to each chemical. The AQI Only Model fitted a quadratic discriminant analysis model with concentration category as the outcome variable and weighted AQI as the explanatory feature. The HMS Only Model fitted a quadratic discriminant analysis model with concentration category as the outcome variable and HMS Index as the explanatory feature. Discriminant analysis models were fitted using the R package *MASS* v.7.3–58.1 (Venables & Ripley, 2002).

We also constructed a classification tree model with concentration category as the outcome variable and (a) weighted PM_2.5_ AQI (AQI), (b) HMS Index, (c) proportion of time in light smoke density (Light), (d) proportion of time in medium smoke density (Medium), (e) proportion of time in heavy smoke density (Heavy), (f) which of the three seasons the participant was in (Season), (g) distance traveled category (Distance Traveled), and (h) self‐reported time spent indoors category (Time Indoors) were the explanatory features. We refer to a model using these explanatory features as “Standard Model” hereafter. While tree‐based models such as classification trees and random forests have the potential to indirectly model interactions between pairs of variables, it has been shown that their ability to model interactions between variables is highly dependent on the data structure and strength of the predictors' marginal relationship with the response variable (Winham et al., 2013; Wright et al., 2016). Therefore, we fit a classification tree model with concentration category as the outcome variable with interaction variables, explicitly defined between Season and AQI and Season and HMS Index, used in addition to variables in the Standard Model as explanatory features. We refer to a model using both sets of features as an “Interaction Model” hereafter. Classification tree models were fitted using the R package *rpart* v.4.1.16 (Therneau & Atkinson, 2022). All classification trees were pruned to the optimal depth based on cross‐validation error. Variable importance was calculated using the Gini Index (Strobl et al., 2007) which measures the ability of a feature to discriminate observations from different categories conditional on other features used in the model.

We fit all models using 10‐fold cross‐validation. Because the random sampling of observations can affect model outcomes, we implemented cross‐validation over 100 bootstrap repetitions for each model (James et al., 2013). Cross‐validation predictive accuracy was used as the metric of model performance. For each chemical, we used a two‐sample *z*‐test for proportions to determine if the mean accuracy of the Interaction model was significantly greater than the mean accuracy of the Standard Model. If the Interaction Model had a significantly higher mean accuracy, it was used as the final model for a chemical, otherwise the Standard Model was used. We refer to the final model used as the “Multivariate Model” hereafter. For each chemical, we compared any two models' mean accuracies using a two‐sample *z*‐test for a difference in proportions, and we computed confidence intervals for the mean accuracy of a model using a single sample proportion confidence interval.

## 3 Results and Discussion

### 3.1 Wristband Chemical Data

Of the 94 organic chemicals tested for in the 364 wristbands, 69 were detected in at least one wristband. The most frequently detected chemicals above the LOD and without matrix interference were xylenes (*m* and *p*; 98.0%), *n*‐tetradecane (97.5%), and ethylbenzene (96.9%). Among chemical concentrations that were above the LOD, the median chemical concentrations were highest for *n*‐nonane (15.6 log_2_ pmol/g wristband), xylenes (*m* and *p*; 13.0 log_2_ pmol/g wristband), and ethylbenzene (11.9 log_2_ pmol/g wristband). Detection frequency, median, and range information for each chemical is listed in Table A2 of Supporting Information S1.

**Figure 1:**
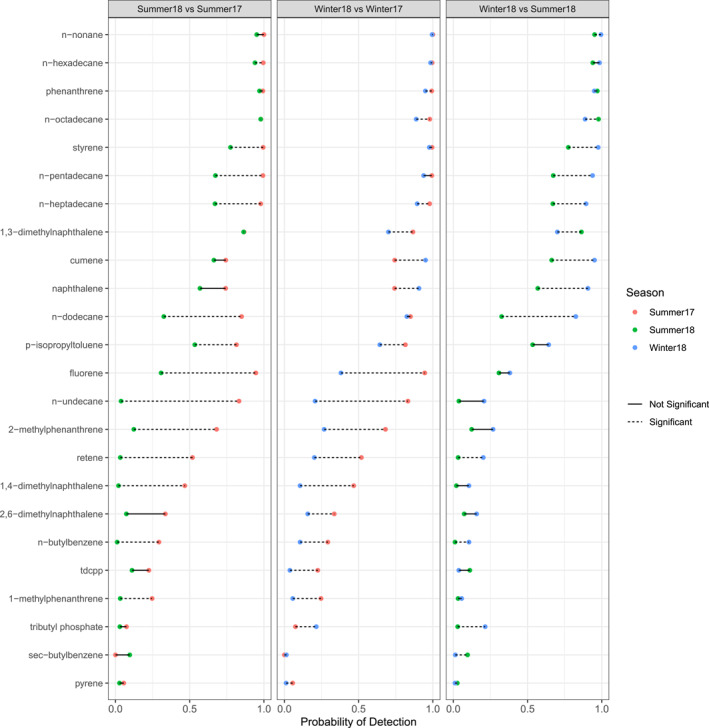
Statistical comparisons of the seasonal detection probability for chemicals with at least one significant seasonal comparison. Probabilities of detection for each season, as estimated by the generalized linear model, are color coded by the season wristbands were worn. Chemicals are ordered by mean detection frequency.

There were 56 chemicals that met the requirements for testing for seasonal differences in detection frequency. Results for the 24 chemicals with at least one significant seasonal comparison are shown in Figure 1.

S17 had higher probabilities of detection for most chemicals compared to the other two seasons (Figure 1). For example, 15 and 14 chemicals had significantly higher probabilities of detection in S17 compared to W18 and S18, respectively. Seven chemicals also had significantly higher mean concentrations in S17 compared to W18 (Table A4 in Supporting Information S1). Four chemicals had significantly higher mean concentrations in S17 compared to S18. Nine chemicals had significantly higher mean concentrations in S18 compared to S17. Three chemicals (tributyl phosphate, naphthalene, and cumene) had consistently significantly higher probabilities of detection in W18 compared to both summers. No chemicals had significantly higher mean concentrations in both summers compared to the winter.

This study happened to coincide with two periods of wildfire smoke, one in S17 and one in S18. In S17, 34 wristbands were worn when a participant's weighted PM_2.5_ AQI was higher than 100 (unhealthy for sensitive groups). In S18, three wristbands were worn when a participant's weighted PM_2.5_ AQI was higher than 100. Eight chemicals had a significant relationship between detection probability in the wristbands and HMS and PM_2.5_ AQI variables (Table 2).

**Table 2:** Chemicals With a Significant Relationship Between Detection Probability and Hazard Mapping System and PM_2.5_ Air Quality Index Variables With Direction of Association Listed

Chemical in wristband	HMS index direction (*p*‐value)	HMS category direction (*p*‐value)			PM_2.5_ AQI direction (*p*‐value)
		Heavy	Medium	Light	
TCDPP	Positive (5e−4)				Positive (5e−3)
Cumene	Negative (2e−4)	Negative (4e−4)			
Fluorene	Positive (4e−3)		Positive (7e−3)		
*p*‐isopropyltoluene	Positive (6e−3)				
1‐methylphenanthrene	Positive (1e−2)			Positive (2e−2)	
1,2‐dimethylnaphthalene	Positive (1e−2)				
*n*‐undecane			Positive (4e−2)	Positive (2e−2)	
2‐methylphenanthrene				Positive (2e−2)	

*Note. P*‐values for tests of significance are in parentheses.

The results of calculating Spearman's correlations between wristband concentrations for each chemical and HMS and PM_2.5_ AQI are found in Table A5 of Supporting Information S1. The correlation values reveal very weak relationships (AQI correlations range from −0.27 to 0.22; HMS correlations range from −0.30 to 0.27). In many cases, higher values of PM_2.5_ AQI values were correlated with lower concentrations in the wristbands. These correlation results indicate that the personal chemical exposure cannot be solely explained by PM_2.5_ AQI or HMS data alone.

Some of the chemical differences in detection probability and mean chemical concentrations between seasons could be due to wildfire smoke in the Eugene, Oregon area during the summers from wildland‐urban interface fires. The chemical differences could be from the chemicals present in the smoke itself or from changes in personal behavior due to the smoke (e.g., spending more time indoors). Many of the chemicals listed in Figure 1 and Table A4 in Supporting Information S1 have been found in wildfire smoke in previous research. For example, PAHs are formed during incomplete burning of organic substances and are found in wildfire smoke; PAHs such as naphthalene, 1‐methylnaphthalene, 2‐methylnaphthalene, 2,6‐dimethylnaphthalene, fluorene, fluoranthene, phenanthrene, and 2‐methylphenanthrene (Rager et al., 2021). In addition, *n*‐tetradecane, *n*‐pentadecane, *n*‐hexadecane, and *n*‐octadecane are alkanes (hydrocarbon chains) that have been detected in wildfire smoke (Rager et al., 2021). It is important to note that, like all chemicals, these chemicals can come from a variety of different sources and not just from wildfires.

Our study took place during a period of heavy wildfire smoke from around 2 September 2017 to 5 September 2017. As stated by a local news article on 3 September 2017, “The Lane Regional Air Protection Agency has detected a hazardous level of air quality in Eugene…All residents are encouraged to stay inside and keep windows closed” (KVAL, 2017). The University of Oregon in Eugene issued a public safety alert on 4 September 2017, further describing the intensity of the smoke: “Wildfires around the Willamette Valley and recent weather have combined to bring severe smoke to the area…Local smoke has been so extreme that some [University of Oregon] air handling systems interpret it as a building fire” (Resnick, 2017).

Our study also captured wildfire smoke in mid‐August of S18. A Eugene newspaper wrote on 20 August 2018, “Air quality in Lane County deteriorated over several hours Monday…as winds continued to funnel smoke from wildfires in Canada and Washington state south…This forecast means the area is likely to endure the worst air quality since wildfire smoke put the Eugene‐Springfield in a chokehold with purple, or very unhealthy, air quality for three consecutive days in early September 2017” (Hill, 2018).

The wildfire smoke in S17 was likely different from S18 due to a variety of factors including differences in smoke density, distance traveled, smoke age, meteorological conditions, and original material/foliage burned. For example, the wildfire smoke in S18 traveled from farther away (Canada and Washington) than the smoke traveled in S17 (Oregon). Research has demonstrated that the chemical composition of smoke is dependent in part on the original fuel source (Rager et al., 2021; Samburova et al., 2016; Urbanski et al., 2008). A recent study has also found that chemical mixtures from different wildfire fuel types have different toxicities in terms of pulmonary immune and injury markers in mice (Rager et al., 2021). The wildfire differences between S17 and S18 along with the differences we capture in wristband concentrations and probabilities of detection between S17 and S18 (Figure 1 and Table A4 in Supporting Information S1) highlight the need for more research in order to better understand how wildfire smoke characteristics influence personal chemical exposure.

### 3.2 GPS Data From Phones

Participants traveled a median distance of 15.4 miles (0.0–264.3 miles) with an interquartile range of 31 miles (75th percentile–25th percentile = 35.2–4.2 miles). Total distance traveled was less than one mile for 18.1% of the 364 wristband sampling periods.

Participants tended to travel less in winter than summer. The median distance traveled was 18.8 miles in S17, 13.1 miles in W18, and 17.2 miles in S18. Participants traveled significantly less in W18 compared to S17 (*p* = 0.014). There was not a significant difference between distance traveled in W18 and S18 (*p* = 0.712).

**Figure 2:**
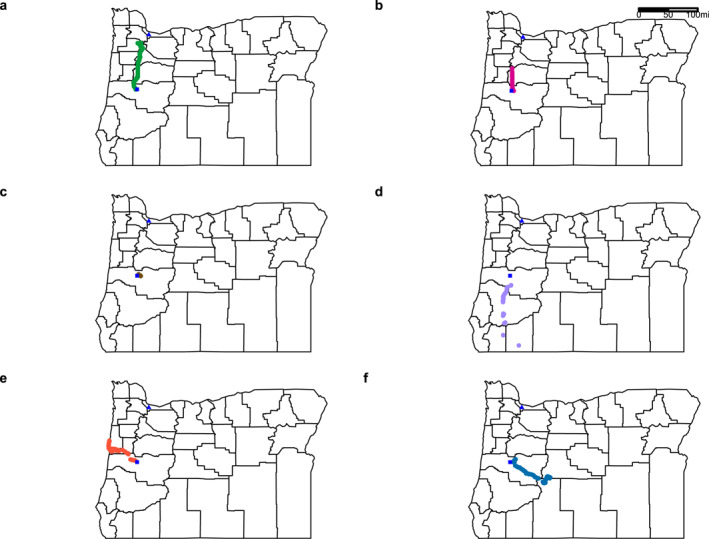
Example global positioning system data for a single wristband sampling period for six participants with different travel patterns where a participant (a) traveled north, (b) traveled to another neighboring county, (c) did not travel to another county, (d) traveled south, (e) traveled west to the Pacific Ocean coast, and (f) traveled east to central Oregon. The blue triangle represents Portland, Oregon and the blue square represents Eugene, Oregon.

Participants traveled in a variety of different directions throughout this study (Figures 2a–2f). For example, some participants traveled north toward Portland, Oregon (Figure 2a), while others remained near home in Eugene, Oregon (Figure 2c).

### 3.3 Behavioral Data From Questionnaires

Roughly half of the participants reported that they spent a majority of their time indoors. Of 364 daily questionnaires, 51.9% reported 12–24 hr spent indoors, 27.2% reported 9–12 hr spent indoors, 18.7% reported 1–8 hr indoors, and 1.9% reported less than one hour spent indoors. The distributions of answers to the questionnaire on time spent indoors by season are shown in Figure A5 of Supporting Information S1. Questionnaires indicate that more time was spent indoors in S18 compared to the two other seasons. For example, 68%, 41%, and 56% of questionnaires reported 12–24 hr indoors in S18, S17, and W18, respectively.

Outdoor temperature may be part of the reason for these differences. For example, the mean heat index based on daily maximum temperature was higher in S18 than S17 for days of our study (92.1°F in S18 and 83.6°F in S17; Figure A6 in Supporting Information S1). The average of the daily maximum temperature for days of our study was 52.3°F in W18, 78.2°F in S17, and 85.4°F in S18.

### 3.4 PM_2.5_ AQI Data

Out of the 49 PM_2.5_ monitors in Oregon in 2017 and 2018, we used information from 23 monitors for further data analysis, representing the monitors closest to the participant's GPS locations during the study (Figure A3 in Supporting Information S1). PM_2.5_ monitors are not spread uniformly across the state, which can be a limitation of using this type of data for personal chemical exposure. For example, we did not have PM_2.5_ data from monitors located on the Oregon Coast during the study period (Figure A3 in Supporting Information S1) yet we do know that at least one participant traveled to the Oregon Coast (Figure 2e). In this instance, we had to use PM_2.5_ AQI data from the closest monitor, which was located on the other side of the coastal mountain range and may not be representative of the true PM_2.5_ AQI that the person experienced.

However, a strength of this study is that nearly all participants were close to PM_2.5_ monitors. Across all matched GPS and PM_2.5_ AQI measurements, PM_2.5_ monitors were a median distance of 2.52 miles away from GPS observations. Ninety percent of GPS observations were within 13.36 miles of the closest PM_2.5_ monitor. At most, someone was 49.34 miles away from the closest PM_2.5_ monitor. This particular case is the aforementioned example of a participant traveling to the Oregon Coast when there was wildfire smoke in S17.

**Figure 3:**
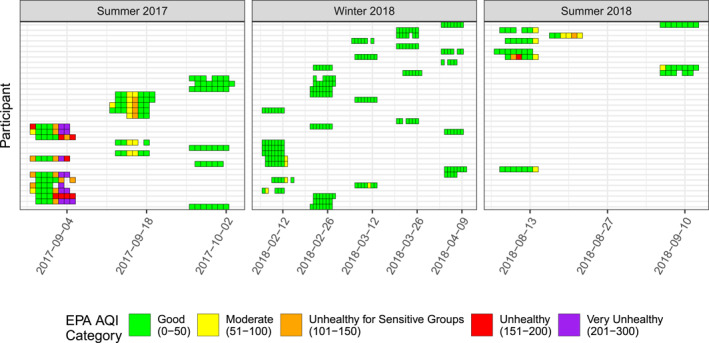
PM_2.5_ air quality index category per wristband sampling period by participant over the three seasons.

The weighted PM_2.5_ AQI value for each wristband sampling period by participant is shown in Figure 3. We used the standard U.S. EPA AQI categories for visualization purposes. In general, S17 had higher PM_2.5_ AQI values than S18. This matches trends in the 2022 Oregon Department of Environmental Quality (DEQ) Wildfire Smoke Trends Report (DEQ, 2022).

The weighted PM_2.5_ AQI mean value was significantly different between all seasons (S18 vs. S17 *p*‐value = 0.002; S17 vs. W18 *p*‐value = 1.18e−10; S18 vs. W18 *p*‐value = 6.72e−5; Figure A7 in Supporting Information S1). The mean weighted PM_2.5_ AQI was 65.1 for S17 [standard deviation (s) = 73.0; maximum value (max) = 292.0], 41.0 for S18 (*s* = 31.0, max = 160.0), and 21.6 for W18 (*s* = 13.4, max = 62.4).

### 3.5 HMS Data

**Figure 4:**
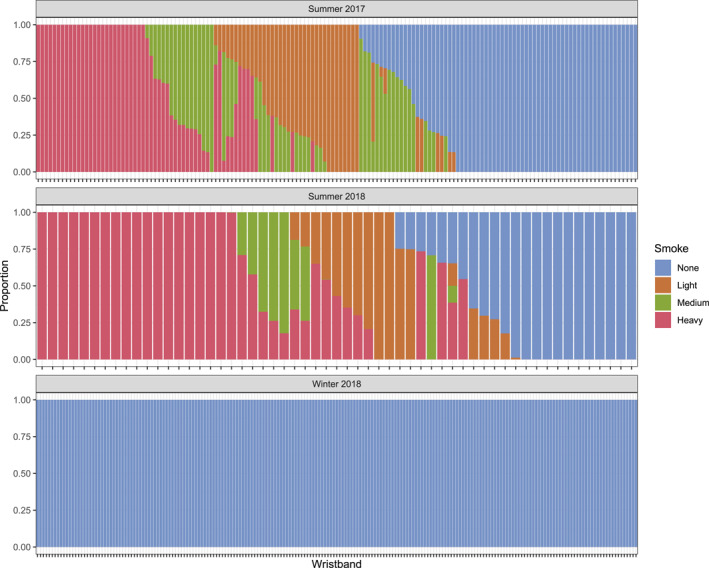
Proportion of time spent in each Hazard Mapping System category by season. Each bar on the *x*‐axis represents one wristband in the respective sampling period.

Overall, 62.6% of wristband sampling periods were located in an area with no smoke coverage according to HMS (Figure 4). By season, 30.2%, 100%, and 22.0% of wristband sampling periods were located in an area with no smoke coverage in S17, W18, and S18, respectively. Further, 11.0% of wristband sampling periods were located in an area with heavy smoke all day according to HMS. By season, 18.0%, 0%, and 30.0% of wristband sampling periods were located in an area with heavy smoke coverage in S17, W18, and S18, respectively.

The HMS Index distribution for the two summers look different with participants in S18 having more values toward the ends of the metric scale rather than the middle when compared to S17 (Figure A8 in Supporting Information S1). The median HMS Index value was 0.61 and 0.42 for S18 and S17, respectively. The median difference in typical HMS index values for S18 compared to S17 were nearly statistically significant (p‐value = 0.051). The differences in HMS Index values between the two summers could be due to many variables such as differences in wind patterns, cloud cover, and type and size of wildfire.

We also compared the weighted PM_2.5_ AQI data with HMS Index values for each wristband sampling period to better understand the relationship between these two proxies for outdoor chemical exposure, and we found that the weighted PM_2.5_ AQI data and HMS scores contain different information (Figure A9 in Supporting Information S1). Specifically, the Spearman's correlation between the two variables is 0.629. Our results are in alignment with other research studies that have compared PM_2.5_ values and HMS values. For example, a study in California found a weak relationship between HMS smoke levels and PM_2.5_ monitoring data from 2007 to 2013 (Preisler et al., 2015). Further, on days where fire plumes were observed with the HMS, the researchers report that the increase in PM_2.5_ at monitors is imprecise (Preisler et al., 2015). Another study in California during the 2017–2018 wildfire seasons concluded that HMS data and PM_2.5_ monitoring data characterized air pollution differently (Fadadu et al., 2020).

### 3.6 Modeling

**Figure 5:**
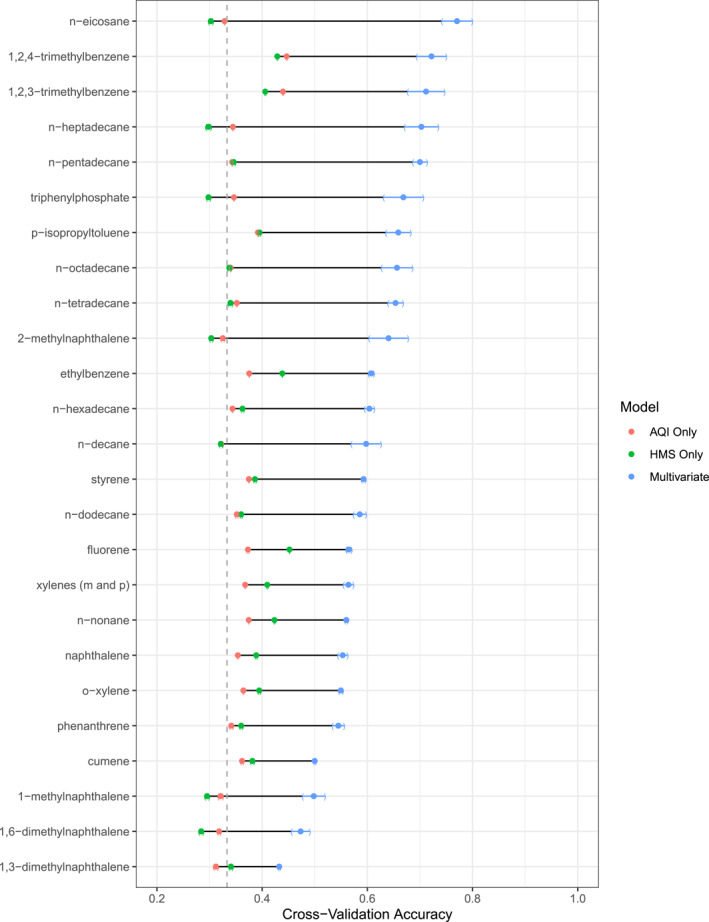
Results from cross‐validation model performance in predicting wristband exposure tertiles (Low, Medium, and High) over one hundred bootstrap iterations. Air quality index (AQI) Only indicates classification tree models fit using PM_2.5_ AQI as the only predictor variable. Hazard Mapping System (HMS) Only indicates classification tree models fit using HMS as the only predictor variable. Multivariate indicates classification tree models fit using PM_2.5_ AQI, HMS, and additional behavioral and environmental variables. Points represent mean accuracy for each model's classification of low, medium, and high concentrations. Parentheses represent 95% confidence intervals for the mean classification accuracy. Chemicals are ordered by the best model accuracy. The dotted vertical line is the average cross‐validation accuracy from a non‐informative model based on random predictions (0.33).

We compared the predictive performance of the models based on PM_2.5_ AQI or HMS only (AQI Model or HMS Model) to the model using additional behavioral and environmental variables (Multivariate Model). Additionally, because there are three categories of wristband concentrations (Low, Medium, and High), we used the predictive performance of a non‐informative model based on random predictions (0.33 accuracy on average) as a baseline performance metric. The AQI Model performed better than random chance for 20 of the 25 chemical models (80%; Figure 5). However, the maximum cross‐validation accuracy was 0.447 for 1,2,4‐trimethylbenzene with the AQI Model. The HMS Model performed better than random chance for 18 of the 25 chemical models (72%). However, the maximum cross‐validation accuracy was 0.452 for fluorene with the HMS Model.

The Interaction Model (model including all variables and interactions between PM_2.5_ AQI and HMS with season) performed best compared to the Standard Model (no interaction variables specified) for 15 out of the 25 chemical models, but only four of those had significantly higher mean accuracies: o‐xylene, *n*‐nonane, phenanthrene, and 1,6‐dimethylnaphthalene (Table A3 in Supporting Information S1). Notably, phenanthrene had a mean accuracy that was higher by 0.076 for the Interaction Model compared to the Standard Model (0.545 compared to 0.469). The resulting final Multivariate Models were predominantly composed of standard variables without explicit specification of interaction variables.

In all cases, the Multivariate Model performed significantly better than the AQI Model and HMS Model (Figure 5 and Table A3 in Supporting Information S1). The maximum cross‐validation accuracy was 0.770 for *n*‐eicosane with the Multivariate Model.

On average, the Multivariate Model increased predictive accuracy by 70.2% (25%–155% improvement depending on the chemical) compared to HMS Model. On average, the Multivariate Model increased predictive accuracy by 70.1% (38.2%–134.5% depending on the chemical) compared to the AQI Model. Table A3 in Supporting Information S1 reports the difference in mean model prediction accuracy for each chemical.

The Multivariate Model performance in Figure 5 indicates that PM_2.5_ AQI and HMS, which are typically used as proxies for air quality, are not predictive of personal chemical exposure and other variables must be considered. The improvement in predictive performance when using the Multivariate Models indicates that the use of PM_2.5_ AQI and HMS, though convenient and readily available, are not sufficient proxies for personal chemical exposure alone. Future studies should include the use of personalized exposure measurement tools, such as silicone wristbands, when possible. Alternatively, significant research investigating the effects of behavior (e.g., time spent in transit) as they relate to personal chemical exposure is needed so questionnaires can supplement convenient proxy measures of exposure.

The interpretation of classification tree models and the determination of which features are most important is a complex task. However, for each Multivariate Model, we can examine which variables are driving the predictive performance of a model by looking at variable importance. Figure A10 in Supporting Information S1 shows the variable importance for all chemicals where the Multivariate Model had a cross‐validation mean prediction accuracy significantly greater than 0.50 (23 of the 25 chemicals modeled). Variable importance value magnitudes are dependent on the data set. Therefore, we examine scaled variable importance values by dividing by the maximum variable importance for each chemical. Variables that are never used in constructing the optimal classification tree have variable importance values of zero. This may be because the variable provides little to no predictive value given the outcome and other variables considered, or the variable is highly correlated with another feature that has better predictive value. Features used in the model will have variable importance values greater than zero. In general, features with higher variable importance values are more important to the model's predictive efficacy. However, this is conditional on other variables included in the model, and any variable with a non‐zero importance value adds predictive value to the model.

**Figure 6:**
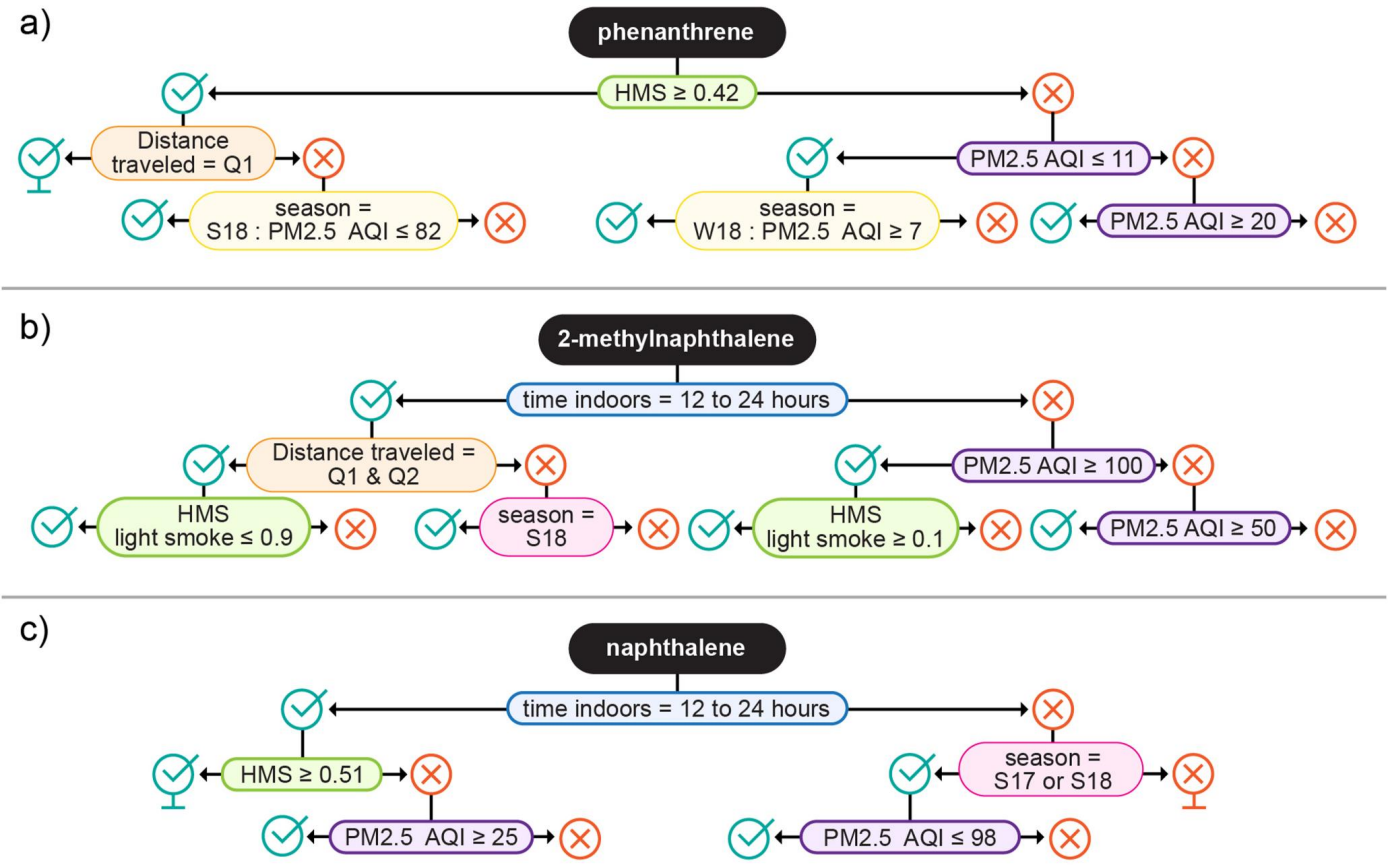
The first few splits of the classification tree models for three PAHs: (a) phenanthrene, (b) 2‐methylnaphthalene, and (c) naphthalene.

We further examined the structure of the classification trees built for the 23 chemicals. Each classification tree is complex, and the placement of variables is quite different between chemicals. Figure 6 includes an example classification tree from the 100 bootstrap iterations for three PAHs (naphthalene, 2‐methylnaphthalene, and phenanthrene). We show the top of the tree such that branches were not split more than three times for these examples. For naphthalene, time indoors and PM_2.5_ AQI equally had the highest variable importance scores (Figure A10 in Supporting Information S1). For 2‐methylnaphthalene and phenanthrene, PM_2.5_ AQI was the variable with the highest importance score (Figure A10 in Supporting Information S1). However, the classification trees did not only depend on the PM_2.5_ AQI variable (Figure 6). For instance, in the top of the phenanthrene example tree in Figure 6a, although the PM_2.5_ AQI variable is used in several places, it is included in two places as an interaction variable dependent on season and other variables are also used in the tree (distance traveled and HMS; Figure 6c).

There are other general patterns to note. *N*‐octadecane (an alkane) had some of the most complex classification trees. Classification trees revealed generally higher *n*‐octadecane (an alkane) wristband concentrations when there were higher AQI values and higher HMS index values. Participants that spent more time indoors (12–24 hr) also tended to have higher *n*‐octadecane wristband concentrations. Wristbands worn during S17 tended to have higher *n*‐octadecane concentrations as well. There were higher wristband concentrations of *n*‐heptadecane (another alkane) in S17 compared to the other seasons. Regardless of season, there were higher wristband concentrations of *n*‐heptadecane when the PM_2.5_ AQI values were higher. There is limited research on sources of long‐chain alkanes that contribute to personal chemical exposure. A recent study detected various alkanes including *n*‐octadecane and *n*‐heptadecane in wildfire smoke, and found that *n*‐octadecane was significantly correlated with a biological endpoint (a lung injury marker at 24 hr; Rager et al., 2021). Certain plants regularly emit alkanes (Peñuelas & Llusià, 2004; Song et al., 2007). Alkanes are also constituents of petroleum products. More research on personal exposure to alkanes is warranted given the high probabilities of detections in wristbands (Figure 1), high wristband concentrations (Table A2 in Supporting Information S1), and classification tree trends related to season, PM_2.5_ AQI values, and HMS smoke index values.

The classification trees and mean variable importance values revealed trends related to the distance traveled variable for a subset of chemicals. Wristbands worn while traveling can capture chemicals in materials within vehicles (like flame retardants in polyurethane foam) and outdoor air pollutants generated by vehicles or road construction (like components of diesel exhaust). For example, the triphenyl phosphate classification trees showed that participants that had a travel distance in the lowest quartile had lower triphenyl phosphate wristband concentrations. People can be exposed to triphenyl phosphate in a variety of ways; this chemical is used as a plasticizer, a lubricant, and a component of commonly used commercial flame retardant formulations (Phillips et al., 2017; van der Veen and de Boer, 2012). Recent research indicates that flame retardants are found in vehicles and time spent traveling in a vehicle can lead to increased personal exposure to some flame retardants in certain cases (Reddam et al., 2020). Specifically, wristbands were worn during commutes and authors found that triphenyl phosphate was strongly correlated with other flame retardants and that longer commutes were associated with increased exposure to one flame retardant, tris(1,3‐dichloro‐2‐propyl)phosphate (Reddam et al., 2020). More research is needed to better understand how vehicle conditions, such as vehicle age, air temperature, and time spent in a vehicle, influences personal chemical exposure.

Traveling in a vehicle may result in exposure of additional chemicals beyond flame retardants, which warrants further investigation. In the *n*‐nonane (an alkane) classification trees, generally the participants that were in the highest quartile for distance traveled had higher wristband concentrations. *N*‐nonane exposure can be from a variety of sources, but research in Asia indicates that *n*‐nonane exposure can be found in diesel vehicle exhaust and roadway asphalt application (Y. Liu et al., 2008). In addition, in the styrene (a VOC) classification trees, the participants that were in the highest quartile for distance traveled in all seasons had higher wristband concentrations. Styrene is found in automobile exhaust (ATSDR, 2012). Styrene wristband concentrations also were higher in winter compared to the two summers. In winter, there were generally higher styrene wristband concentrations when participants spent more time indoors (12–24 hr indoors). These patterns also may be partially explained by common sources of styrene such as building materials, cigarette smoke, and some industrial facility emissions (ATSDR, 2012). In our study, participants' travel habits (i.e., total distance traveled) did not associate with smoke or air quality conditions.

### 3.7 Strengths and Limitations

To our knowledge, this is the largest wristband data set (over 40,000 chemical concentrations) used to investigate the predictive value of variables on personal chemical exposure. This study is strengthened by the inclusion of repeated daily measures over a 7‐day period from participants across different seasons. This enabled us to have a well‐powered study where we could leverage repeated measures to quantify and include within person variability in modeling. However, the 7‐day period may not fully capture the range of an individual's exposure throughout an entire season.

Another strength of this study is the use of the ELF tool, which allowed us to collect paired wristbands, daily questionnaire responses, and GPS observations for each participant during the study period with high compliance rates. This study adds to a growing collection of studies using the ELF tool (Evoy et al., 2022; Rohlman et al., 2019). Future studies can continue to develop and use the ELF tool to easily gather robust data sets about personal behaviors and/or health outcomes related to chemical exposure. In our study, variables collected by the ELF tool's App (distance traveled and time indoors) contributed important information to the multivariate models (Figure A10 in Supporting Information S1).

A strength of this study is the use of wristbands and wristband‐related analytical methods which allows us to generate a large chemical data set for each study participant that is not possible with PM_2.5_ air monitors. For example, we detected 69 unique chemicals in wristbands, which provided additional insight into personal chemical exposure. Another benefit of wristbands over stationary PM_2.5_ air monitors is that they easily travel with a person, such as when a person is driving in a car, cooking in the kitchen, or running outside.

A limitation of this study is that the data may not be representative of other summers and winters in Eugene, Oregon. This study only covers specific time periods in S17, S18, and W18. Wildfire smoke can differ greatly from year to year (DEQ, 2022). However, a strength of this study is that we were able to capture data during periods of heavy wildfire smoke in S17 and S18 (Figure 4). Understanding personal chemical exposure before, during, and after wildfire smoke is critical since the frequency and duration of wildfires in the western U.S. has increased since the mid‐1980s (Westerling et al., 2006) and wildfire smoke is a complex mixture of chemicals that can adversely affect health, especially for sensitive groups, such as asthmatics (Aguilera, Corringham, Gershunov, Leibel, & Benmarhnia, 2021; Arbex et al., 2007; Chen et al., 2021; EPA, 2021).

Participant demographics in this study are not representative of the overall U.S. population. Here, participants were all asthmatics and primarily white college‐educated females that did not identify as Hispanic or Latino (Table 1). In addition, participants had the time and interest to volunteer to be in the study.

Interpretation of the questionnaire data in our study is limited due to the broad time categories in the available answers and, as with any questionnaire data, there is potential for the question to be misinterpreted by participants. However, it is widely cited that Americans spend most of their time indoors; specifically, a national U.S. phone survey from 1992 to 1994 found that respondents reported spending an average of 87% of their time in enclosed buildings (Klepeis et al., 2001), and our results generally align with this data (Section 3.3).

We did not expect complete agreement between the information collected by the wristbands and the outdoor proxies for chemical exposure (PM_2.5_ AQI from stationary monitors and HMS from satellites) because they inherently capture different information. For instance, wristbands capture chemicals available to be absorbed in the body and are not samplers of PM_2.5_. Although chemicals adsorbed to the surface of PM_2.5_ that come in contact with the wristband may partition into the silicone similarly how they would partition into the body. Regardless, our Multivariate Models that included information from PM_2.5_ stationary monitors and HMS satellites resulted in good accuracy (Figure 5).

## 4 Conclusions

While PM_2.5_ AQI data from stationary air monitors and HMS data are commonly used in epidemiological studies as proxies for personal chemical exposure, our results demonstrate that PM_2.5_ AQI or HMS data alone are insufficient to explain complex personal chemical exposures. Future research is needed to determine if there are additional environmental and behavioral factors that can be combined with stationary PM_2.5_ data and HMS data to better predict personal chemical exposure. Researchers can also use personal samplers, such as silicone wristbands, to assess personal chemical exposure to a wide variety of organic chemicals.

We observed seasonal differences for organic chemical detection and concentration. For the variables we collected, these differences appear to be due to a variety of factors and not necessarily due to differences between people because we have repeated measures for each participant. Our data indicate that behaviors (e.g., time spent indoors and distance traveled) as well as the presence of wildfire smoke could be influencing personal organic chemical exposure. Future studies should ideally span multiple seasons.

Additional variables could be collected from participants to achieve better predictive power of personal chemical exposure in the future. Examples of such variables include information on building type, air filtration, and ventilation. Another example would be more detailed information about time indoors and outdoors on a finer time scale. In addition, it may be helpful to collect the specific amounts of time spent indoors at a workplace, a vehicle, and home separately.

## Conflict of Interest

Kim A. Anderson and Diana Rohlman, authors of this research, disclose a financial interest in MyExposome, Inc., which is marketing products related to the research being reported. The terms of this arrangement have been reviewed and approved by Oregon State University in accordance with its policy on research conflicts of interest. The authors have no other relevant financial or non‐financial interests to disclose.

## Supporting information

Supporting Information S1

## Data Availability

Non‐personally identifiable wristband data for participants that provided consent for their data to be shared are available at Bramer et al. (2023).
