# Supplementary material for: PM2.5 Is Insufficient to Explain Personal PAH Exposure
Source: Geohealth. 2024 Feb 10;8(2):e2023GH000937. doi: 10.1029/2023GH000937 (PMC10858395; doi:10.1029/2023GH000937)
Supplement: Supplementary file 1 — Supporting Information S1 [file GH2-8-e2023GH000937-s001.pdf]

*GeoHealth*

Supporting Information for

**$PM_{2.5}$ is Insufficient to Explain Personal PAH Exposure**

**Lisa M. Bramer[1*], Holly M. Dixon[2*], Diana Rohlman[3], Richard P. Scott[2]; Rachel L. Miller[4]; Laurel Kincl[3]; Julie B. Herbstman[5]; Katrina M. Waters[1,2]; Kim A. Anderson[2]**

[*]These coauthors contributed equally

[1] Pacific Northwest National Laboratory, Biological Sciences Division, Richland, Washington, U.S.

[2]Oregon State University, Environmental and Molecular Toxicology, Food Safety and Environmental Stewardship Program, Corvallis, Oregon, U.S.

[3]Oregon State University, College of Health, Corvallis, Oregon, U.S.

[4]School of Medicine at Mount Sinai, Division of Clinical Immunology, New York City, New York, U.S.

[5]Columbia University, Columbia Center for Children's Environmental Health, Department of Environmental Health Sciences, Mailman School of Public Health, New York City, New York, U.S.

Corresponding author: Kim A. Anderson (kim.anderson@oregonstate.edu)

## Contents of this file

**Table A1** Study participant demographics by season ........ 3
**Fig. A1** Summary information for the wristband, GPS, questionnaire, $PM_{2.5}$ AQI, and HMS datasets included in this study........ 4
**Text A1 Wristband Methodology** ........ 5
**Table A2** Summary information statistics for the 94 volatile and semi-volatile organic chemicals (VOCs and SVOCs) target analytes for the 364 wristbands used in modeling ........ 6
**Text A2 GPS Methodology** ........ 10
**Fig. A2** The number of observed GPS locations for each hour of the day before and after the application of the movement rate filter ........ 11
**Fig. A3** Map of 49 $PM_{2.5}$ monitors in Oregon in 2017 and 2018 ........ 12
**Fig. A4** Plot of example HMS data from September 15, 2017 ........ 13
**Table A3** Model performance comparisons........ 14
**Table A4** Seasonal comparison of mean chemical concentrations ........ 15
**Table A5** Spearman's correlations between wristband concentrations for each chemical and HMS and $PM_{2.5}$ AQI ........ 16
**Fig. A5** Distributions of answers to questionnaire on time spent indoors by season ........ 17
**Fig. A6** Distributions of daily maximum heat index in summer 2017 and summer 2018........ 18
**Fig. A7** Distributions of time weighted $PM_{2.5}$ AQI by season ........ 19
**Fig. A8** HMS Index distributions for summer 2017 and summer 2018 ........ 20
**Fig. A9** Comparison of the time-weighted $PM_{2.5}$ AQI with HMS scores from the same wristband sampling period ........ 21
**Fig. A10** Heatmap of variable importance scores from each chemical's modeling results ........ 22

## Additional Supporting Information

Non-personally identifiable wristband data for participants that provided consent for their data to be shared are publicly available and can be found on Pacific Northwest National Laboratory's DataHub (https://data.pnnl.gov/group/nodes/dataset/33707).

**Table A1** Study participant demographics by summer 2017 (S17), winter 2018 (W18), and summer 2018 (S18).

| | S17 (*n* = 23) | | W18 (*n* = 32) | | S18 (*n* = 9) | |
|---|---|---|---|---|---|---|
| Continuous Characteristic | Range | Mean ± SD | Range | Mean ± SD | Range | Mean ± SD |
| Age (years) | 28-70 | 51±13 | 21-74 | 48±15 | 21-72 | 41±15 |
| | | | | | | |
| Categorical Characteristics | Number of People | Percent out of 23 | Number of People | Percent out of 32 | Number of People | Percent out of 9 |
| Gender | | | | | | |
| Female | 18 | 78.3 | 27 | 84.4 | 8 | 88.9 |
| Male | 5 | 21.7 | 5 | 15.6 | 1 | 11.1 |
| Education Level | | | | | | |
| High School | 1 | 4.4 | 1 | 3.1 | 0 | 0.0 |
| Some College | 4 | 17.4 | 6 | 18.8 | 2 | 22.2 |
| Associate’s Degree | 4 | 17.4 | 5 | 15.6 | 1 | 11.1 |
| Bachelor’s Degree | 9 | 39.1 | 10 | 31.2 | 3 | 33.3 |
| Master’s Degree | 4 | 17.4 | 8 | 25.0 | 3 | 33.3 |
| Doctoral Degree | 0 | 0.0 | 1 | 3.1 | 0 | 0.0 |
| Prefer Not to Answer | 1 | 4.4 | 1 | 3.1 | 0 | 0.0 |
| Household Income | | | | | | |
| Less than $39,999 | 8 | 34.8 | 14 | 43.8 | 5 | 55.6 |
| $40,000 to $79,999 | 7 | 30.4 | 10 | 31.2 | 2 | 22.2 |
| More than $80,000 | 7 | 30.4 | 6 | 18.8 | 1 | 11.1 |
| Prefer Not to Answer | 1 | 4.4 | 2 | 6.2 | 1 | 11.1 |

**Fig. A1** Summary information for the wristband, GPS, questionnaire, $PM_{2.5}$ AQI, and HMS datasets included in this study. Additional details on each dataset are in the methods section. A sampling period is an approximate 24-hour time window when a participant wore a wristband.

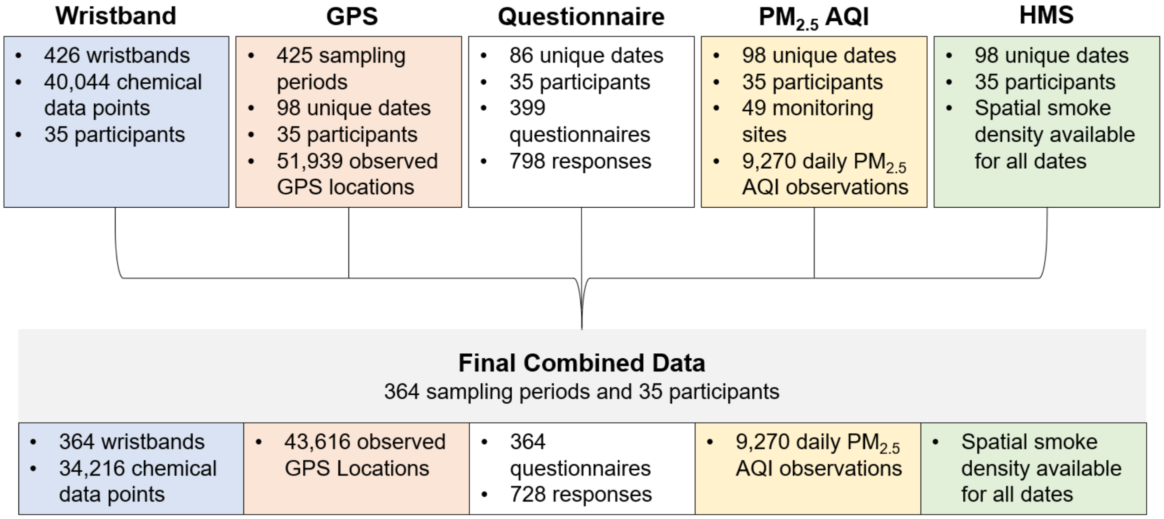

**Text A1 Wristband Methodology**

***Additional laboratory materials***

We cleaned our equipment and glassware in the laboratory by (1) rinsing with solvent or (2) using a three-step process (i.e., wash in dishwasher with detergent, rinse with 18 MΩ*cm water, and bake for 12 hours at >300°C). We produced 18 MΩ*cm water by filtering water through an Advantage A10 (Merck KGaA,Dramstadt, Germany). We used optima-grade solvents or equivalent from Fisher Scientific (Pittsburgh, PA, US).

Target analyte standards are from

- Sigma-Aldrich (St. Louis, MO, US)
- Accustandard (New Haven, CT, US)
- TCI America (Tokyo, Japan)
- SantaCruz Biotechnology (Dallas, TX, US)
- Chiron (Trondheim, Norway)

***Wristband deployment***

For deployment, wristbands were individually packaged in airtight polytetrafluoroethylene (PTFE) bags (Welch Fluorocarbon, Dover, NH, U.S.). We instructed participants not to apply lotions or other personal care products directly on the wristband.

***Wristband QC***

To ensure data quality and account for potential chemical contamination, we utilized a series of quality control checks throughout wristband preparation, transportation, cleaning, extraction, and analysis processes, as previously described (Dixon et al. 2018; Dixon et al. 2022). In this study, we collected and analyzed blank wristbands that traveled to and from Eugene, Oregon in PTFE bags. We also analyzed wristbands that were never deployed but went through one or all laboratory processes. We averaged and subtracted detected concentrations in 24 QC samples from sample concentrations. QC samples were below the LOD for 67 of the 87 chemicals.

During chemical analysis, we analyzed instrument blanks and calibration verifications (CVs) every 10 to 15 wristband extracts, which accounts for over 15% of the samples run on the instrument. All target chemicals were below the instrument LODs in the hexane or ethyl acetate instrument blanks. To achieve our data quality objectives, we verified that ≥80% of the target compounds were ±20% of the known value in the CV before sample analysis.

**Table A2** Summary information and statistics for the 94 volatile and semi-volatile organic chemicals (VOCs and SVOCs) target analytes for the 364 wristbands used in modeling. The Chemical Abstracts Service (CAS) number, molecular weight (g/mol), limit of detection (LOD), limit of quantitation (LOQ), wristband detection frequency, and summary statistics ($log_2$ pmol/g wristband) are provided for all target analytes in this method. Analytes are listed in order of increasing molecular weight.

| | Target Analyte | CAS Number | Molec-ular Weight (g/mol) | Instrument LOD ($log_2$ pmol/g wristband) | Instrument LOQ ($log_2$ pmol/g wristband) | Number of Wristbands Without Matrix Interference[a] | Detection Frequency (%)[b] | Median ($log_2$ pmol/g wristband) | Range ($log_2$ pmol/g wristband) | Interquartile Range ($log_2$ pmol/g wristband) |
|---|---|---|---|---|---|---|---|---|---|---|
| 1 | styrene | 100-42-5 | 104.1 | 0.45 | 2.8 | 352 | 94 | 11.7 | <LOD - 13.7 | 1.15 |
| 2 | ethylbenzene | 100-41-4 | 106.2 | 0.66 | 3.0 | 352 | 97 | 11.9 | <LOD - 13.6 | 0.923 |
| 3 | o-xylene | 95-47-6 | 106.2 | 0.74 | 3.1 | 352 | 96 | 11.6 | <LOD - 14.1 | 0.952 |
| 4 | xylenes (m and p) | 108-38-3 & 106-42-3 | 106.2 | 0.59 | 2.9 | 352 | 98 | 13.0 | <LOD - 15.5 | 0.931 |
| 5 | chlorobenzene | 108-90-7 | 112.6 | 0.60 | 2.9 | 352 | 3 | 9.60 | <LOD - 11.5 | 1.44 |
| 6 | 1,2,3-trimethylbenzene | 526-73-8 | 120.2 | 0.37 | 2.7 | 364 | 58 | 5.98 | <LOD - 8.26 | 1.57 |
| 7 | cumene | 98-82-8 | 120.2 | 0.35 | 2.7 | 352 | 77 | 9.49 | <LOD - 11.5 | 0.851 |
| 8 | n-propylbenzene | 103-65-1 | 120.2 | 0.33 | 2.7 | 364 | 43 | 5.68 | <LOD - 7.87 | 2.03 |
| 9 | 1,2,4-trimethylbenzene | 95-63-6 | 120.2 | 0.35 | 2.7 | 364 | 54 | 8.30 | <LOD - 10.4 | 1.70 |
| 10 | 1,3,5-trimethylbenzene | 108-67-8 | 120.2 | 0.33 | 2.7 | 364 | 47 | 6.50 | <LOD - 9.01 | 1.66 |
| 11 | 2-chlorotoluene | 95-49-8 | 126.6 | 0.27 | 2.6 | 364 | 0.0 | -- | -- | -- |
| 12 | 4-chlorotoluene | 106-43-4 | 126.6 | 0.23 | 2.6 | 364 | 0.3 | 4.27 | <LOD - 4.27 | -- |
| 13 | naphthalene | 91-20-3 | 128.2 | -2.7 | -0.32 | 364 | 71 | 4.96 | <LOD - 9.02 | 2.29 |
| 14 | n-nonane | 111-84-2 | 128.3 | 0.72 | 3.1 | 352 | 96 | 15.6 | < LOD - 17.9 | 1.22 |
| 15 | n-butylbenzene | 104-51-8 | 134.2 | 0.23 | 2.6 | 364 | 22 | 4.59 | <LOD - 7.11 | 1.98 |
| 16 | p-isopropyltoluene | 99-87-6 | 134.2 | 0.19 | 2.5 | 364 | 65 | 5.39 | <LOD - 12.7 | 1.89 |
| 17 | sec-butylbenzene | 135-98-8 | 134.2 | 0.19 | 2.5 | 364 | 4.9 | 2.28 | <LOD - 4.01 | 1.27 |
| 18 | tert-butylbenzene | 98-06-6 | 134.2 | 0.23 | 2.6 | 364 | 4.1 | 6.16 | <LOD - 9.05 | 1.58 |
| 19 | 1-methylnaphthalene | 90-12-0 | 142.2 | -2.6 | -0.19 | 364 | 96 | 4.69 | <LOD - 7.55 | 1.49 |
| 20 | 2-methylnaphthalene | 91-57-6 | 142.2 | -2.4 | -0.09 | 364 | 97 | 5.57 | <LOD - 9.11 | 1.22 |
| 21 | n-decane | 124-18-5 | 142.3 | 0.14 | 2.5 | 364 | 67 | 7.47 | <LOD - 10.6 | 2.94 |
| 22 | chromone | 491-38-3 | 146.1 | 3.5 | 5.9 | 361 | 0.0 | -- | -- | -- |
| 23 | 1,3-dichlorobenzene | 541-73-1 | 147.0 | -0.01 | 2.3 | 364 | 0.0 | -- | -- | -- |
| 24 | o-dichlorobenzene | 95-50-1 | 147.0 | 0.04 | 2.4 | 364 | 0.3 | 2.12 | <LOD - 2.12 | -- |
| 25 | p-dichlorobenzene | 106-46-7 | 147.0 | 2.3 | 4.7 | 364 | 24 | 3.55 | <LOD - 9.05 | 1.70 |

| Target Analyte | | CAS Number | Molecular Weight (g/mol) | Instrument LOD ($log_2$ pmol/g wristband) | Instrument LOQ ($log_2$ pmol/g wristband) | Number of Wristbands Without Matrix Interference[a] | Detection Frequency (%)[b] | Median ($log_2$ pmol/g wristband) | Range ($log_2$ pmol/g wristband) | Interquartile Range ($log_2$ pmol/g wristband) |
|---|---|---|---|---|---|---|---|---|---|---|
| 26 | acenaphthylene | 208-96-8 | 152.2 | -2.4 | -0.15 | 364 | 0.0 | -- | -- | -- |
| 27 | acenaphthene | 83-32-9 | 154.2 | -2.4 | -0.16 | 364 | 4.4 | 3.97 | <LOD - 6.39 | 2.02 |
| 28 | 1,2-dimethylnaphthalene | 573-98-8 | 156.2 | -2.7 | -0.43 | 364 | 8.8 | 3.78 | <LOD - 5.47 | 1.61 |
| 29 | 1,4-dimethylnaphthalene | 571-58-4 | 156.2 | -2.6 | -0.30 | 364 | 30 | 4.30 | <LOD - 6.41 | 0.969 |
| 30 | 1,5-dimethylnaphthalene | 571-61-9 | 156.2 | -2.1 | 0.28 | 364 | 1.6 | 3.72 | <LOD - 5.32 | 0.936 |
| 31 | 1,6-dimethylnaphthalene | 575-43-9 | 156.2 | -2.8 | -0.49 | 364 | 86 | 4.88 | <LOD - 6.70 | 0.967 |
| 32 | 1,8-dimethylnaphthalene | 569-41-5 | 156.2 | -2.7 | -0.41 | 364 | 0.0 | -- | -- | -- |
| 33 | 2,6-dimethylnaphthalene | 581-42-0 | 156.2 | -2.7 | -0.41 | 364 | 31 | 4.81 | <LOD - 7.19 | 1.11 |
| 34 | 1,3-dimethylnaphthalene | 575-41-7 | 156.2 | -2.7 | -0.33 | 364 | 73 | 4.95 | <LOD - 7.11 | 0.901 |
| 35 | n-undecane | 1120-21-4 | 156.3 | 0.03 | 2.4 | 364 | 47 | 9.12 | <LOD – 14.0 | 1.35 |
| 36 | bromobenzene | 108-86-1 | 157.0 | -0.15 | 2.2 | 364 | 0.0 | -- | -- | -- |
| 37 | fluorene | 86-73-7 | 166.2 | -2.1 | 0.27 | 364 | 60 | 3.83 | <LOD - 7.10 | 1.33 |
| 38 | n-dodecane | 112-40-3 | 170.3 | 0.02 | 2.3 | 364 | 71 | 7.10 | <LOD - 11.5 | 1.59 |
| 39 | anthracene | 120-12-7 | 178.2 | -0.22 | 2.1 | 360 | 4.7 | 4.58 | <LOD - 7.53 | 1.21 |
| 40 | phenanthrene | 85-01-8 | 178.2 | -2.9 | -0.62 | 360 | 95 | 5.70 | <LOD - 9.19 | 1.02 |
| 41 | 9-fluorenone | 486-25-9 | 180.2 | -3.0 | -0.75 | 361 | 1.1 | 4.49 | <LOD - 6.06 | 1.31 |
| 42 | 1,2,3-trichlorobenzene | 87-61-6 | 181.4 | -0.19 | 2.1 | 364 | 0.3 | 2.14 | <LOD - 2.14 | -- |
| 43 | 1,2,4-trichlorobenzene | 120-82-1 | 181.4 | -0.21 | 2.1 | 364 | 5.2 | 2.84 | <LOD - 3.86 | 1.21 |
| 44 | dibenzothiophene | 132-65-0 | 184.3 | -0.97 | 1.4 | 364 | 1.9 | 4.46 | <LOD - 6.41 | 0.299 |
| 45 | 1-methylphenanthrene | 832-69-9 | 192.3 | -0.21 | 2.1 | 360 | 18 | 4.01 | <LOD - 6.44 | 0.980 |
| 46 | 2-methylanthracene | 613-12-7 | 192.3 | 2.3 | 4.6 | 360 | 2.2 | 5.10 | <LOD - 9.66 | 0.593 |
| 47 | 2-methylphenanthrene | 2531-84-2 | 192.3 | -3.0 | -0.68 | 360 | 42 | 4.36 | <LOD - 6.28 | 1.08 |
| 48 | 9-methylanthracene | 779-02-2 | 192.3 | 3.0 | 5.3 | 360 | 0.0 | -- | -- | -- |
| 49 | xanthone | 90-47-1 | 196.2 | -1.1 | 1.2 | 361 | 0.6 | 9.02 | <LOD - 9.67 | 0.652 |
| 50 | n-tetradecane | 629-59-4 | 198.4 | -0.15 | 2.2 | 364 | 98 | 10.0 | <LOD - 14.5 | 1.36 |
| 51 | fluoranthene | 206-44-0 | 202.3 | -3.2 | -0.83 | 355 | 13 | 3.79 | <LOD - 8.31 | 0.975 |
| 52 | pyrene | 129-00-0 | 202.3 | -2.8 | -0.51 | 355 | 15 | 3.88 | <LOD - 7.90 | 0.731 |
| 53 | 2,3-dimethylanthracene | 613-06-9 | 206.3 | 2.9 | 5.2 | 355 | 0.0 | -- | -- | -- |
| 54 | 3,6-dimethylphenanthrene | 1576-67-6 | 206.3 | -0.66 | 1.7 | 360 | 1.9 | 5.75 | <LOD - 6.39 | 1.88 |
| 55 | 9,10-anthraquinone | 84-65-1 | 208.2 | 3.0 | 5.3 | 361 | 1.1 | 6.89 | <LOD - 7.52 | 0.228 |
| 56 | n-pentadecane | 629-62-9 | 212.4 | -0.25 | 2.1 | 364 | 91 | 10.2 | <LOD - 14.7 | 1.53 |

| | Target Analyte | CAS Number | Molecular Weight (g/mol) | Instrument LOD ($log_2$ pmol/g wristband) | Instrument LOQ ($log_2$ pmol/g wristband) | Number of Wristbands Without Matrix Interference[a] | Detection Frequency (%)[b] | Median ($log_2$ pmol/g wristband) | Range ($log_2$ pmol/g wristband) | Interquartile Range ($log_2$ pmol/g wristband) |
|---|---|---|---|---|---|---|---|---|---|---|
| 57 | 1-methylpyrene | 2381-21-7 | 216.3 | -2.9 | -0.56 | 355 | 1.1 | 2.92 | <LOD - 3.94 | 0.679 |
| 58 | benzo[a]fluorene | 238-84-6 | 216.3 | -2.5 | -0.24 | 355 | 1.4 | 3.09 | <LOD - 5.70 | 0.345 |
| 59 | benzo[b+c]fluorene | 243-17-4 & 205-12-9 | 216.3 | -2.8 | -0.50 | 355 | 0.0 | -- | -- | -- |
| 60 | cyclopenta[cd]pyrene | 27208-37-3 | 226.3 | -0.2 | 2.1 | 355 | 0.0 | -- | -- | -- |
| 61 | n-hexadecane | 544-76-3 | 226.4 | -0.34 | 2.0 | 364 | 94 | 9.88 | <LOD - 13.5 | 1.38 |
| 62 | chrysene | 218-01-9 | 228.3 | 0.73 | 3.1 | 355 | 0.3 | 6.41 | <LOD - 6.41 | -- |
| 63 | triphenylene | 217-59-4 | 228.3 | 0.23 | 2.6 | 355 | 0.0 | -- | -- | -- |
| 64 | benz[a]anthracene | 56-55-3 | 228.3 | 0.20 | 2.5 | 355 | 0.0 | -- | -- | -- |
| 65 | benzanthrone | 82-05-3 | 230.3 | 2.0 | 4.4 | 361 | 0.3 | 4.59 | <LOD - 4.60 | -- |
| 66 | benzofluorenone | 76723-60-9 | 230.3 | 1.8 | 4.1 | 361 | 0.0 | -- | -- | -- |
| 67 | retene | 483-65-8 | 234.3 | -3.9 | -1.5 | 355 | 37 | 3.58 | <LOD - 6.94 | 0.965 |
| 68 | 2-ethylanthraquinone | 84-51-5 | 236.3 | 2.3 | 4.6 | 361 | 0.6 | 5.93 | <LOD - 8.21 | 2.27 |
| 69 | n-heptadecane | 629-78-7 | 240.5 | -0.44 | 1.9 | 364 | 81 | 11.0 | <LOD - 13.7 | 1.63 |
| 70 | 5-methylchrysene | 3697-24-3 | 242.3 | 0.72 | 3.0 | 355 | 0.3 | 3.41 | <LOD - 3.41 | -- |
| 71 | 6-methylchrysene | 1705-85-7 | 242.3 | 0.24 | 2.6 | 355 | 0.0 | -- | -- | -- |
| 72 | benzo[e]pyrene | 192-97-2 | 252.3 | 2.6 | 4.9 | 342 | 0.0 | -- | -- | -- |
| 73 | benzo[a]pyrene | 50-32-8 | 252.3 | -0.41 | 1.9 | 342 | 1.5 | 4.56 | <LOD - 7.23 | 1.26 |
| 74 | benzo[j]fluoranthene | 205-82-3 | 252.3 | 2.7 | 5.1 | 342 | 0.3 | 7.25 | <LOD - 7.25 | -- |
| 75 | benzo[b+k]fluoranthene | 205-99-2 & 207-08-9 | 252.3 | 3.6 | 5.9 | 342 | 0.0 | -- | -- | -- |
| 76 | n-octadecane | 593-45-3 | 254.5 | -0.49 | 1.8 | 364 | 89 | 10.7 | <LOD - 15.9 | 1.32 |
| 77 | tributyl phosphate | 126-73-8 | 266.3 | 1.4 | 3.7 | 361 | 24 | 4.57 | <LOD - 8.52 | 1.25 |
| 78 | anthanthrene | 191-26-4 | 276.3 | 5.6 | 7.9 | 361 | 0.0 | -- | -- | -- |
| 79 | benzo[ghi]perylene | 191-24-2 | 276.3 | -0.70 | 1.6 | 361 | 0.0 | -- | -- | -- |
| 80 | indeno[1,2,3-cd]pyrene | 193-39-5 | 276.3 | 2.3 | 4.6 | 342 | 0.0 | -- | -- | -- |
| 81 | dibenzo[a,h]anthracene | 53-70-3 | 278.3 | 5.8 | 8.1 | 342 | 0.0 | -- | -- | -- |
| 82 | benzo[a]chrysene | 213-46-7 | 278.3 | 5.3 | 7.7 | 342 | 0.3 | 8.52 | <LOD - 8.52 | -- |
| 83 | n-eicosane | 112-95-8 | 282.5 | -0.62 | 1.7 | 364 | 66.5 | 10.7 | <LOD - 15.2 | 1.01 |
| 84 | TCEP | 115-96-8 | 285.5 | 3.3 | 5.6 | 361 | 11.4 | 4.97 | <LOD - 7.51 | 1.70 |
| 85 | coronene | 191-07-1 | 300.4 | 3.4 | 5.7 | 361 | 0.3 | 5.46 | <LOD - 5.46 | -- |
| 86 | dibenzo[a,e]fluoranthene | 5385-75-1 | 302.4 | 3.9 | 6.2 | 361 | 0.0 | -- | -- | -- |

| | Target Analyte | CAS Number | Molecular Weight (g/mol) | Instrument LOD ($log_2$ pmol/g wristband) | Instrument LOQ ($log_2$ pmol/g wristband) | Number of Wristbands Without Matrix Interference[a] | Detection Frequency (%)[b] | Median ($log_2$ pmol/g wristband) | Range ($log_2$ pmol/g wristband) | Interquartile Range ($log_2$ pmol/g wristband) |
|---|---|---|---|---|---|---|---|---|---|---|
| 87 | dibenzo[a,e]pyrene | 192-65-4 | 302.4 | 3.7 | 6.0 | 361 | 0.0 | -- | -- | -- |
| 88 | dibenzo[a,h]pyrene | 189-64-0 | 302.4 | 11 | 13 | 361 | 0.0 | -- | -- | -- |
| 89 | dibenzo[a,i]pyrene | 189-55-9 | 302.4 | 9.3 | 12 | 361 | 0.0 | -- | -- | -- |
| 90 | dibenzo[a,l]pyrene | 191-30-0 | 302.4 | 3.4 | 5.7 | 361 | 0.0 | -- | -- | -- |
| 91 | triphenylphosphate | 115-86-6 | 326.3 | 1.5 | 3.8 | 361 | 92 | 5.32 | <LOD - 11.8 | 1.63 |
| 92 | tricresyl phosphate | 1330-78-5 | 368.4 | 2.3 | 4.6 | 361 | 1.9 | 7.14 | <LOD - 8.02 | 1.03 |
| 93 | TDCPP | 13674-87-8 | 430.9 | 2.7 | 5.1 | 361 | 15 | 6.96 | <LOD - 9.16 | 1.64 |
| 94 | tris(2-ethylhexyl) phosphate | 78-42-2 | 434.6 | 0.58 | 2.9 | 361 | 13 | 7.31 | <LOD - 10.5 | 2.56 |

[a] We were unable to detect a surrogate due to matrix interference in some cases and, therefore, were unable to quantify the target analytes related to the undetected surrogate. We report the number of wristbands for each target analyte that did not have matrix interference.

[b] Detection frequency was calculated by dividing the number of wristbands with the target analyte detected by the number of wristbands in the study without matrix interference.

**Text A2 GPS Methodology**

Some location measurements may be less reliable due to signal obstruction and other factors. Therefore, we calculated the movement rate of each phone, in miles the phone moved per minute, and then applied a movement rate filter to the data to exclude less reliable GPS locations. We filtered out GPS data points that had greater than a two mile per minute rate using a moving window of two observed locations before and after each GPS data point. The formula used for the movement rate filter is included below.

Let $c_i$ denote the set of GPS coordinates converted to Universal Transverse Mercator (UTM) coordinates observed at time point $i$, that is $c_i = (E_i, N_i)$. Further, let $\mathrm{d_{i,j}}$ denote the distance between the $i^{th}$ location and the $j^{th}$ location, approximated using the Euclidean distance between coordinates and then converted to miles. More explicitly,

$$d_{i,j} = \sqrt{\left(E_i - E_j\right)^2 + \left(N_i - N_j\right)^2}.$$

Also, let $t_i$ represent the time at time point $i$ and $t_{i,j}$ represent the time, in minutes, between time point $i$ and time point $j$. Then, the median movement rate at time point $k$ was calculated as:

$$\widetilde{MR} = median\left\{\frac{d_{k,k-2}}{t_{k,k-2}}, \frac{d_{k,k-1}}{t_{k,k-1}}, \frac{d_{k,k+1}}{t_{k,k+1}}, \frac{d_{k,k+2}}{t_{k,k+2}}\right\}.$$

The movement rate filter resulted in 7.3% of GPS observations being excluded. The movement rate filter did not systematically alter patterns in the distribution of the number of observed GPS locations before and after application of the movement rate filter (Fig. A2). Before applying the movement rate filter, the minimum, maximum, and mean number of GPS observations per wristband was 11, 1216, and 126, respectively. After applying the movement rate filter, the minimum, maximum, and mean number of GPS observations per wristband was 11, 1050, and 117, respectively.

**Fig. A2** The number of observed GPS locations for each hour of the day before and after the application of the movement rate filter.

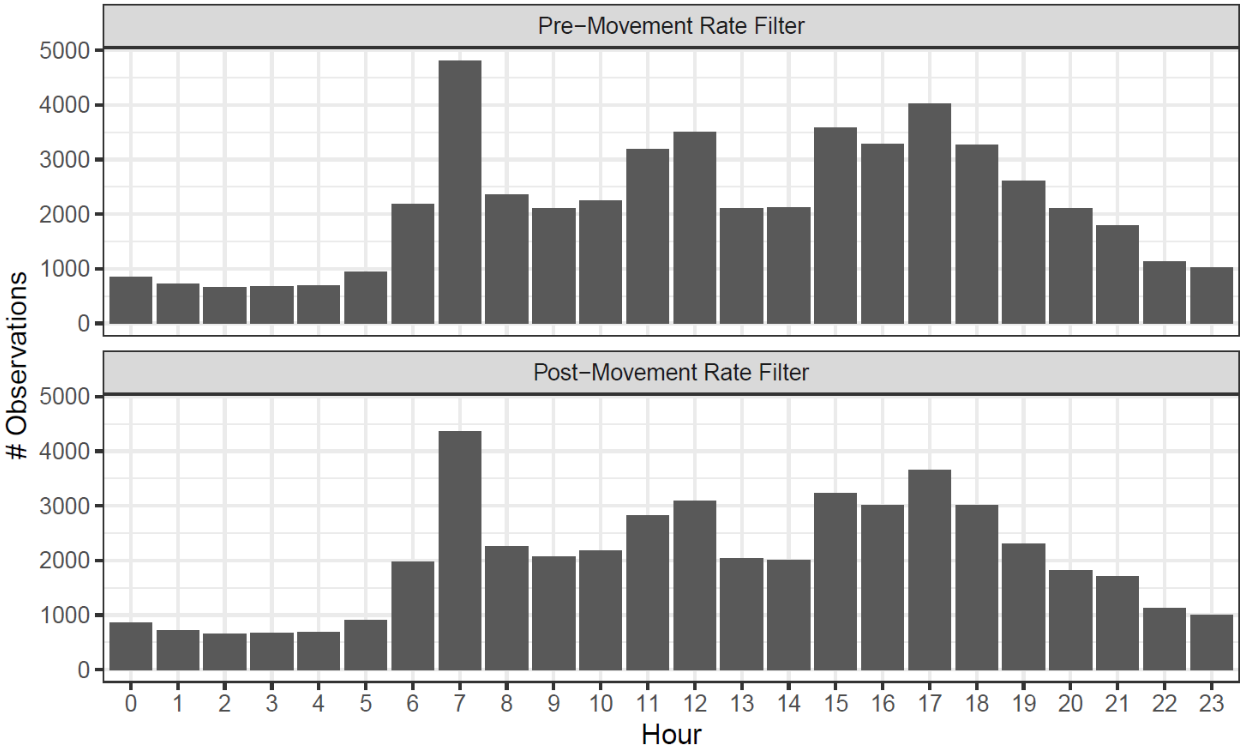

**Fig. A3** Map of 49 $PM_{2.5}$ monitors in Oregon in 2017 and 2018. Blue circles indicate $PM_{2.5}$ monitors that were used in data analysis. Grey circles indicate $PM_{2.5}$ monitors that were not used in data analysis. A red X indicates major Oregon cities.

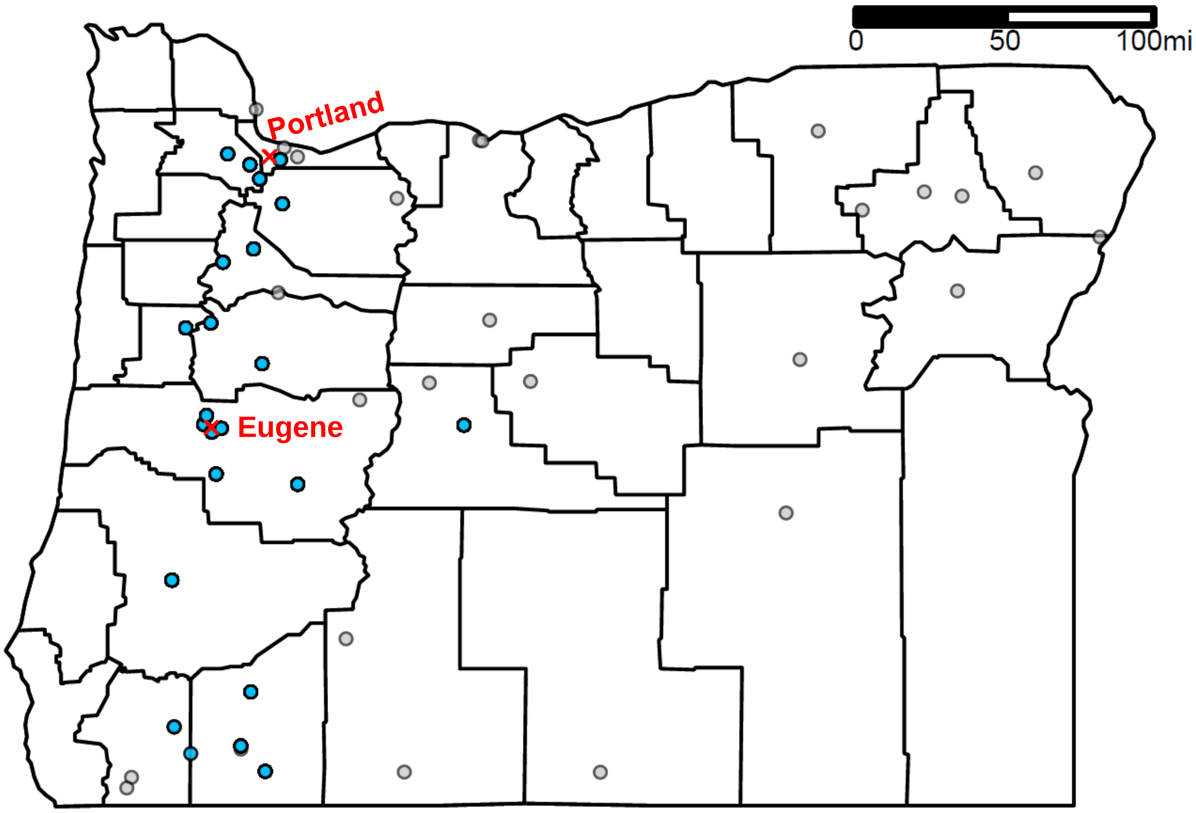

**Fig. A4** Plot of example HMS data from September 15, 2017.

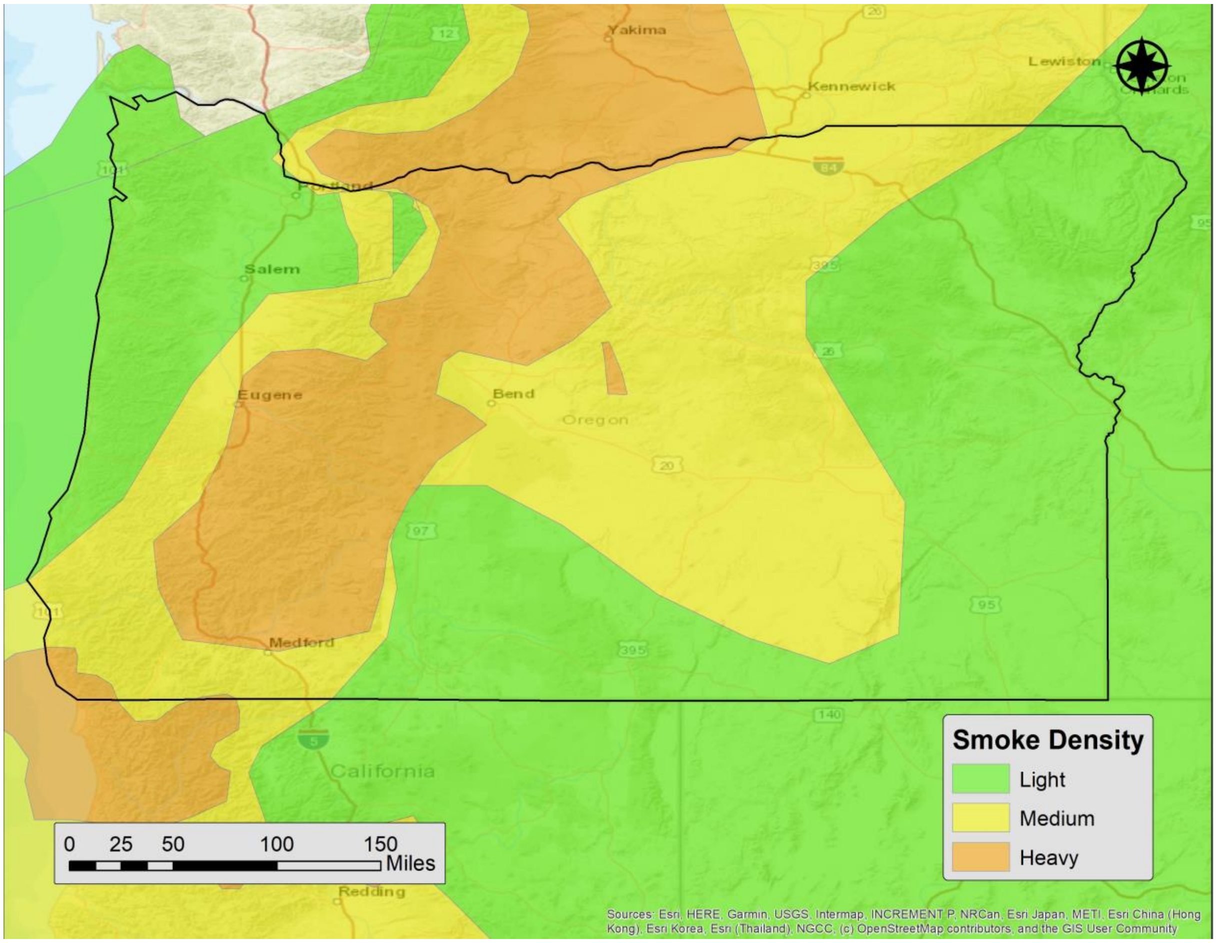

**Table A3** Model performance comparisons. Bold text indicates a significant adjusted p-value of 0.05 or smaller. Chemicals are ordered by mean model accuracy of multivariate compared to the AQI model. Grey shading indicates the first model is significantly higher compared to the second model. Blue shading indicates the second model is significantly higher compared to the first model.

| | Difference in mean model prediction accuracy | | | |
|---|---|---|---|---|
| Chemical | AQI vs HMS (p-value) | Interaction vs Standard (p-value) | Multivariate vs AQI (p-value) | Multivariate vs HMS (p-value) |
| n-eicosane | 0.026 (<1e-20) | -0.116 (3.02e-11) | 0.442 (<1e-20) | 0.468 (<1e-20) |
| n-heptadecane | 0.047 (<1e-20) | -0.015 (0.089) | 0.358 (<1e-20) | 0.405 (<1e-20) |
| n-pentadecane | -0.003 (0.071) | 0.031 (0.126) | 0.357 (<1e-20) | 0.354 (<1e-20) |
| triphenylphosphate | 0.048 (<1e-20) | 0.048 (0.070) | 0.322 (<1e-20) | 0.371 (<1e-20) |
| n-octadecane | 0.002 (0.215) | 0.029 (0.060) | 0.316 (<1e-20) | 0.319 (<1e-20) |
| 2-methylnaphthalene | 0.022 (1.86e-18) | -0.037 (3.78e-4) | 0.315 (<1e-20) | 0.337 (<1e-20) |
| n-tetradecane | 0.012 (3.62e-8) | 0.009 (1.00) | 0.302 (<1e-20) | 0.314 (<1e-20) |
| n-decane | 0.0004 (0.854) | 0.019 (0.693) | 0.276 (<1e-20) | 0.276 (<1e-20) |
| 1,2,4-trimethylbenzene | 0.018 (<1e-20) | 0.013 (0.108) | 0.275 (<1e-20) | 0.293 (<1e-20) |
| 1,2,3-trimethylbenzene | 0.034 (<1e-20) | 0.025 (0.156) | 0.272 (<1e-20) | 0.306 (<1e-20) |
| p-isopropyltoluene | -0.003 (0.005) | 0.015 (0.087) | 0.267 (<1e-20) | 0.264 (<1e-20) |
| n-hexadecane | -0.019 (<1e-20) | -0.006 (3.43e-15) | 0.260 (<1e-20) | 0.241 (<1e-20) |
| n-dodecane | -0.009 (4.62e-6) | 0.021 (0.266) | 0.234 (<1e-20) | 0.225 (<1e-20) |
| ethylbenzene | -0.063 (<1e-20) | -0.006 (1.38e-5) | 0.232 (<1e-20) | 0.169 (<1e-20) |
| styrene | -0.012 (2.69e-14) | -0.001 (0.328) | 0.218 (<1e-20) | 0.207 (<1e-20) |
| phenanthrene | -0.019 (8.21e-15) | 0.076 (<1e-20) | 0.204 (<1e-20) | 0.185 (<1e-20) |
| naphthalene | -0.035 (<1e-20) | -0.021 (4.74e-7) | 0.199 (<1e-20) | 0.164 (<1e-20) |
| xylenes (m and p) | -0.042 (<1e-20) | -0.006 (0.843) | 0.196 (<1e-20) | 0.154 (<1e-20) |
| fluorene | -0.079 (<1e-20) | -0.004 (0.067) | 0.192 (<1e-20) | 0.113 (<1e-20) |
| n-nonane | -0.049 (<1e-20) | 0.007 (3.78e-14) | 0.186 (<1e-20) | 0.137 (<1e-20) |
| o-xylene | -0.030 (<1e-20) | 0.004 (0.007) | 0.185 (<1e-20) | 0.155 (<1e-20) |
| 1-methylnaphthalene | 0.026 (2.71e-16) | 0.0004 (0.773) | 0.177 (<1e-20) | 0.203 (<1e-20) |
| 1,6-dimethylnaphthalene | 0.034 (<1e-20) | 0.010 (0.003) | 0.155 (<1e-20) | 0.189 (<1e-20) |
| cumene | -0.020 (9.72e-18) | -0.003 (0.032) | 0.138 (<1e-20) | 0.118 (<1e-20) |
| 1,3-dimethylnaphthalene | -0.029 (<1e-20) | 0.001 (0.342) | 0.121 (<1e-20) | 0.092 (<1e-20) |

**Table A4** Seasonal comparison of mean chemical concentrations. Chemicals listed have at least one significant comparison. Chemicals are ordered first by the number of significant seasonal comparisons and then the mean of the absolute value of the $\log_2$ fold changes. Color indicates the number of significant seasonal comparisons (blue indicates three, white indicates two, and gray indicates one). An asterisk indicates a significant adjusted p-value of 0.05 or smaller.

| Chemical in Wristband | $\log_2$ fold change of S18 vs. S17 (adjusted p-value) $\log_2 \frac{mean\,S18}{mean\,S17}$ | $\log_2$ fold change of W18 vs. S17 (adjusted p-value) $\log_2 \frac{mean\,W18}{mean\,S17}$ | $\log_2$ fold change of W18 vs. S18 (adjusted p-value) $\log_2 \frac{mean\,W18}{mean\,S18}$ |
|---|---|---|---|
| 1,2,3-trimethylbenzene | **2.37 (1e-8)*** | **0.60 (3e-2)*** | **-1.78 (1e-6)*** |
| cumene | **1.57 (<1e-20)*** | **0.58 (1e-13)*** | **-0.99 (1e-9)*** |
| ethylbenzene | **-0.33 (2e-2)*** | **0.52 (4e-12)*** | **0.85 (2e-13)*** |
| n-hexadecane | **-0.68 (1e-4)*** | **-0.33 (4e-4)*** | **0.35 (4e-2)*** |
| fluoranthene | **2.78 (7e-4)*** | 0.60 (9e-2) | **-2.18 (8e-3)*** |
| n-pentadecane | **-2.16 (4e-16)*** | -0.13 (5e-1) | **2.03 (<1e-20)*** |
| 1,2,4-trimethylbenzene | **1.95 (7e-4)*** | 0.66 (1e-1) | **-1.29 (2e-2)*** |
| n-propylbenzene | **1.46 (8e-4)*** | **0.93 (1e-4)*** | -0.53 (3e-1) |
| 1,3,5-trimethylbenzene | **1.43 (2e-4)*** | 0.19 (6e-1) | **-1.24 (3e-4)*** |
| n-tetradecane | **-0.88 (1e-5)*** | -0.10 (6e-1) | **0.78 (6e-6)*** |
| styrene | -0.09 (9e-1) | **0.63 (2e-9)*** | **0.71 (1e-4)*** |
| xylenes (m and p) | -0.10 (7e-1) | **0.49 (7e-10)*** | **0.58 (1e-6)*** |
| o-xylene | -0.20 (3e-1) | **0.35 (2e-4)*** | **0.55 (1e-4)*** |
| n-undecane | -2.30 (7e-2) | 0.30 (4e-1) | **2.61 (4e-2)*** |
| 1,2,4-trichlorobenzene | **1.68 (5e-2)*** | 1.41 (1e-1) | -0.27 (1e-1) |
| n-decane | **1.41 (6e-3)*** | 0.44 (2e-1) | -0.98 (5e-2) |
| naphthalene | -0.57 (3e-1) | **-0.93 (3e-5)*** | -0.36 (4e-1) |
| fluorene | -0.43 (1e-1) | **-0.69 (1e-8)*** | -0.26 (4e-1) |
| 2-methylphenanthrene | **0.68 (3e-2)*** | 0.15 (4e-1) | -0.53 (8e-2) |
| p-isopropyltoluene | 0.32 (6e-1) | **0.63 (1e-2)*** | 0.31 (6e-1) |
| 1-methylnaphthalene | -0.43 (8e-2) | 0.03 (1e0) | **0.46 (2e-2)*** |
| 2,6-dimethylnaphthalene | 0.17 (1e0) | **-0.24 (4e-2)*** | -0.40 (8e-1) |
| triphenylphosphate | -0.31 (4e-1) | **-0.40 (3e-3)*** | -0.08 (9e-1) |
| phenanthrene | -0.11 (8e-1) | **-0.26 (6e-3)*** | -0.16 (4e-1) |
| 1,3-dimethylnaphthalene | -0.18 (1e-1) | **-0.14 (2e-2)*** | 0.03 (9e-1) |

**Table A5** Spearman's correlations between wristband concentrations for each chemical and HMS and $PM_{2.5}$ AQI. Chemicals are ordered by Spearman's correlation coefficients in the $PM_{2.5}$ AQI comparison.

| Chemical | Spearman's correlation coefficient between: Wristband Concentration and $PM_{2.5}$ AQI | Wristband Concentration and HMS |
|---|---|---|
| fluorene | 0.227 | 0.270 |
| phenanthrene | 0.163 | 0.226 |
| triphenylphosphate | 0.110 | 0.064 |
| n-heptadecane | 0.049 | 0.028 |
| p-isopropyltoluene | 0.049 | 0.162 |
| 1,6-dimethylnaphthalene | 0.039 | 0.030 |
| 1,2,3-trimethylbenzene | 0.022 | 0.079 |
| 1,3-dimethylnaphthalene | 0.019 | 0.132 |
| n-eicosane | 0.010 | -0.034 |
| 1,2,4-trimethylbenzene | 0.008 | 0.068 |
| naphthalene | 0.005 | 0.057 |
| 1-methylnaphthalene | 0.004 | -0.002 |
| 2-methylnaphthalene | -0.008 | 0.042 |
| n-octadecane | -0.034 | 0.055 |
| n-hexadecane | -0.051 | 0.005 |
| n-decane | -0.076 | -0.039 |
| n-tetradecane | -0.107 | -0.054 |
| n-nonane | -0.112 | -0.127 |
| n-pentadecane | -0.121 | -0.108 |
| cumene | -0.128 | -0.218 |
| n-dodecane | -0.142 | -0.142 |
| styrene | -0.145 | -0.300 |
| o-xylene | -0.211 | -0.218 |
| ethylbenzene | -0.212 | -0.344 |
| xylenes (m and p) | -0.266 | -0.302 |

**Fig. A5** Distributions of answers to questionnaire on time spent indoors by season.

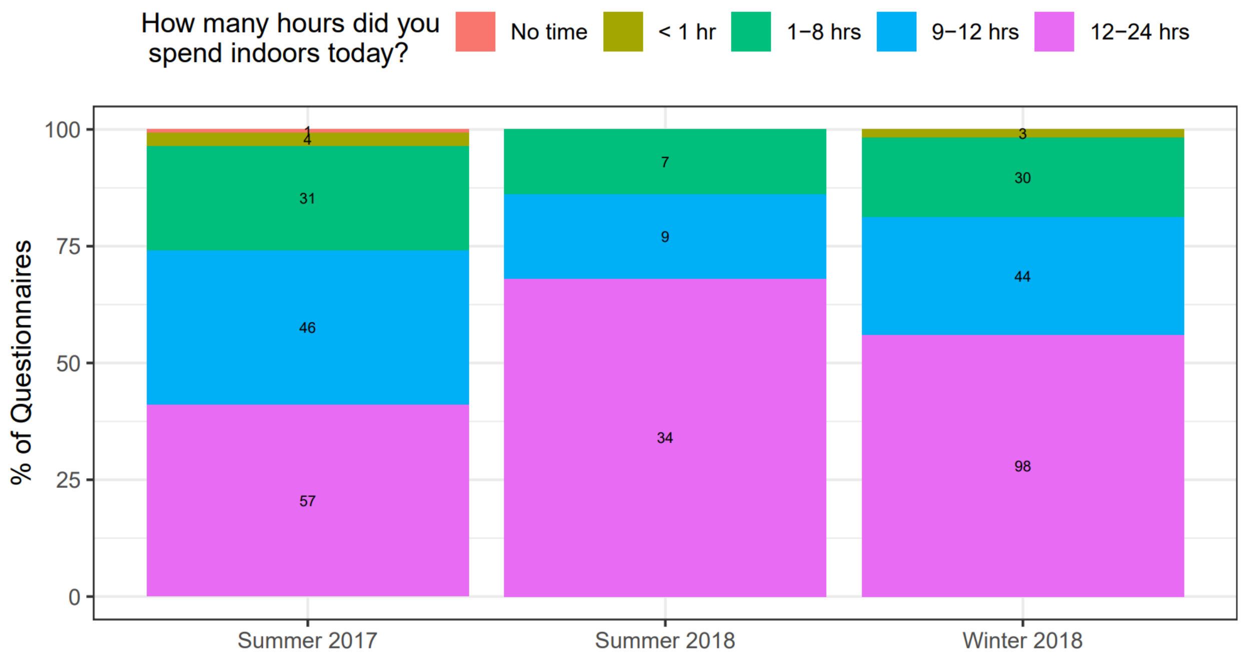

**Fig. A6** Distributions of daily maximum heat index in summer 2017 (S17) and summer 2018 (S18).

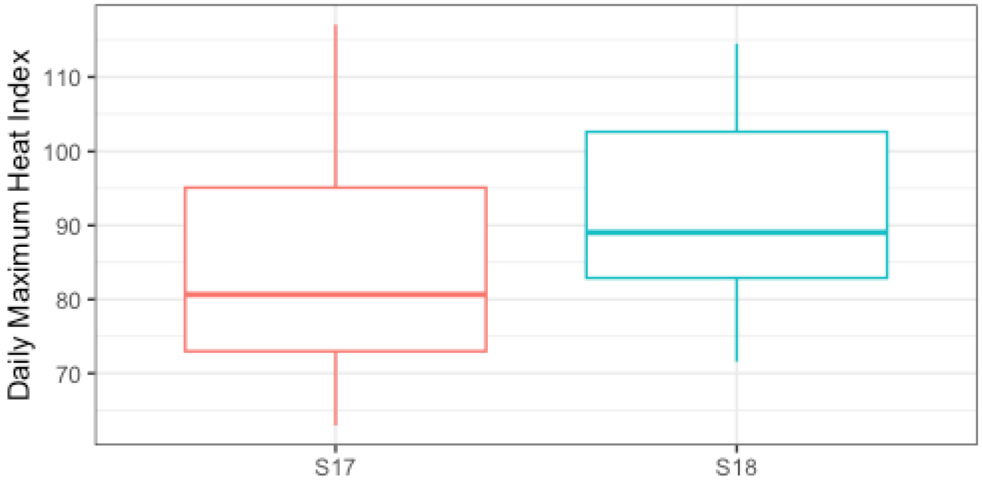

**Fig. A7** Distributions of time weighted $PM_{2.5}$ AQI by season.

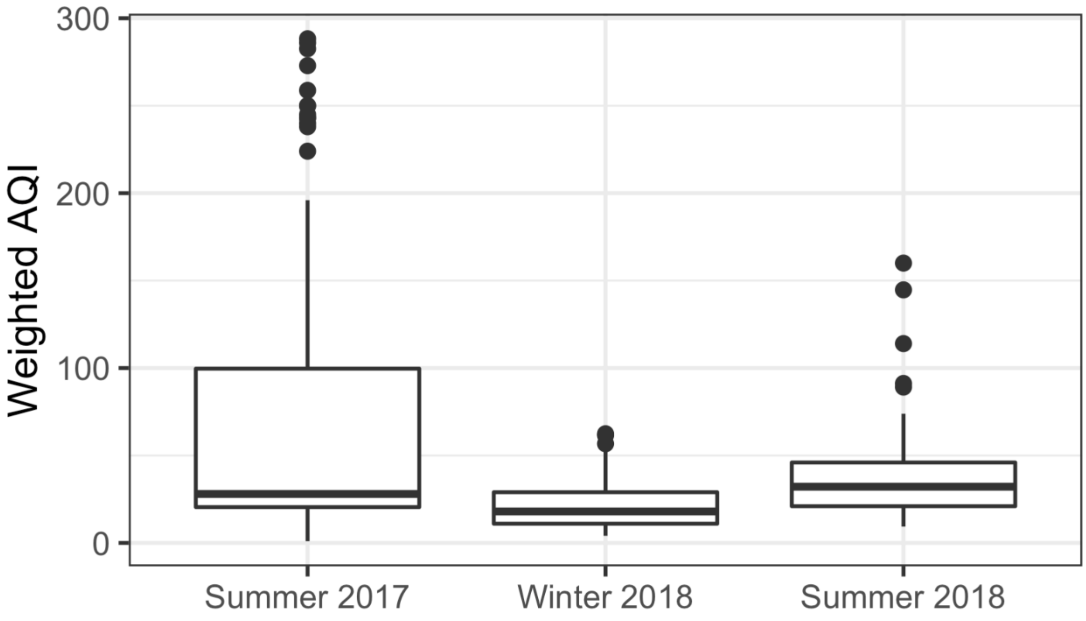

**Fig. A8** HMS Index distributions for summer 2017 and summer 2018.

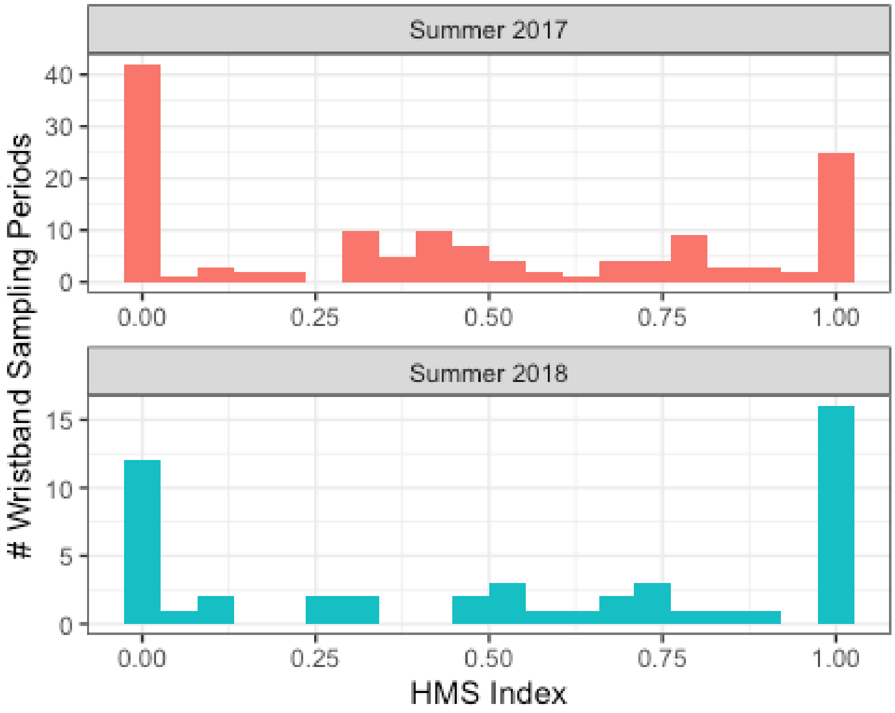

**Fig. A9** Comparison of the time-weighted $PM_{2.5}$ AQI with HMS scores from the same wristband sampling period.

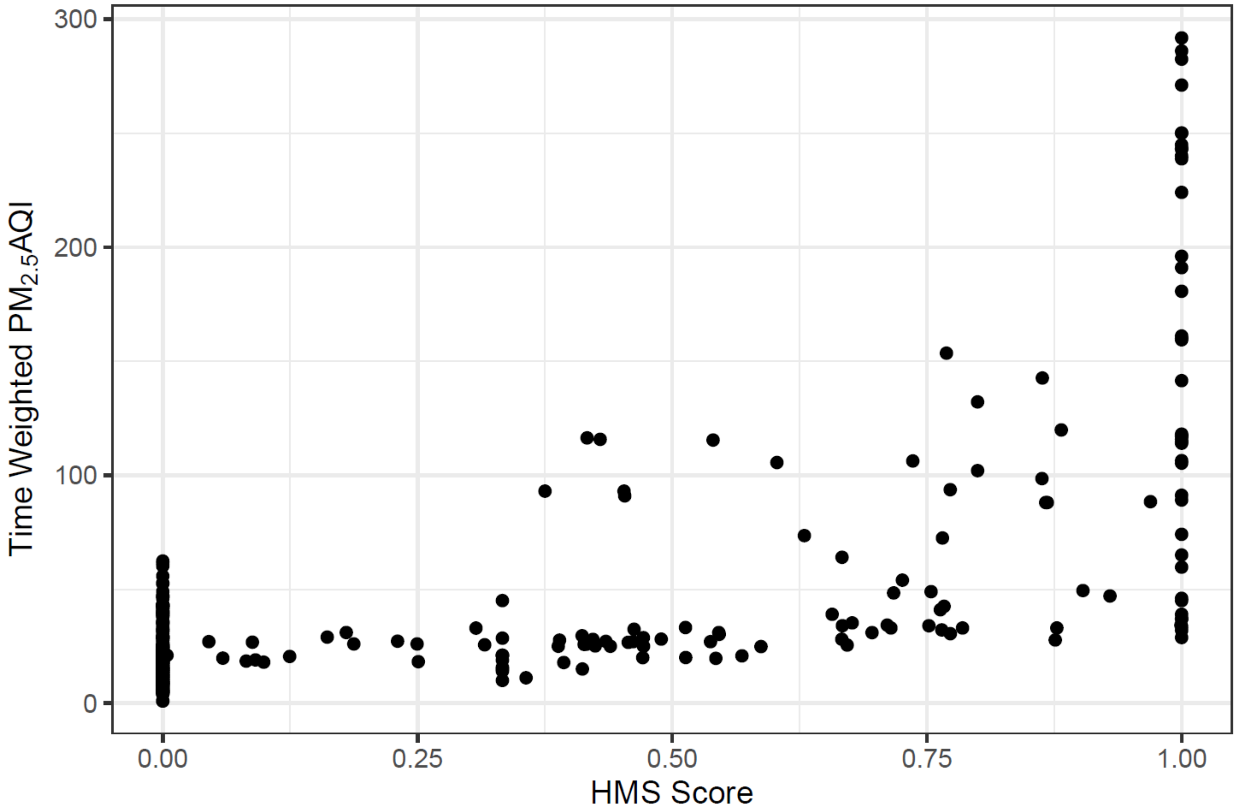

**Fig. A10** Heatmap of variable importance scores from each chemical's modeling results. Results are displayed for models with cross-validated $R^2$ of 0.5 or higher. Chemicals are ordered from highest $R^2$ to lowest. Variables are ordered based on mean variable importance across all models.

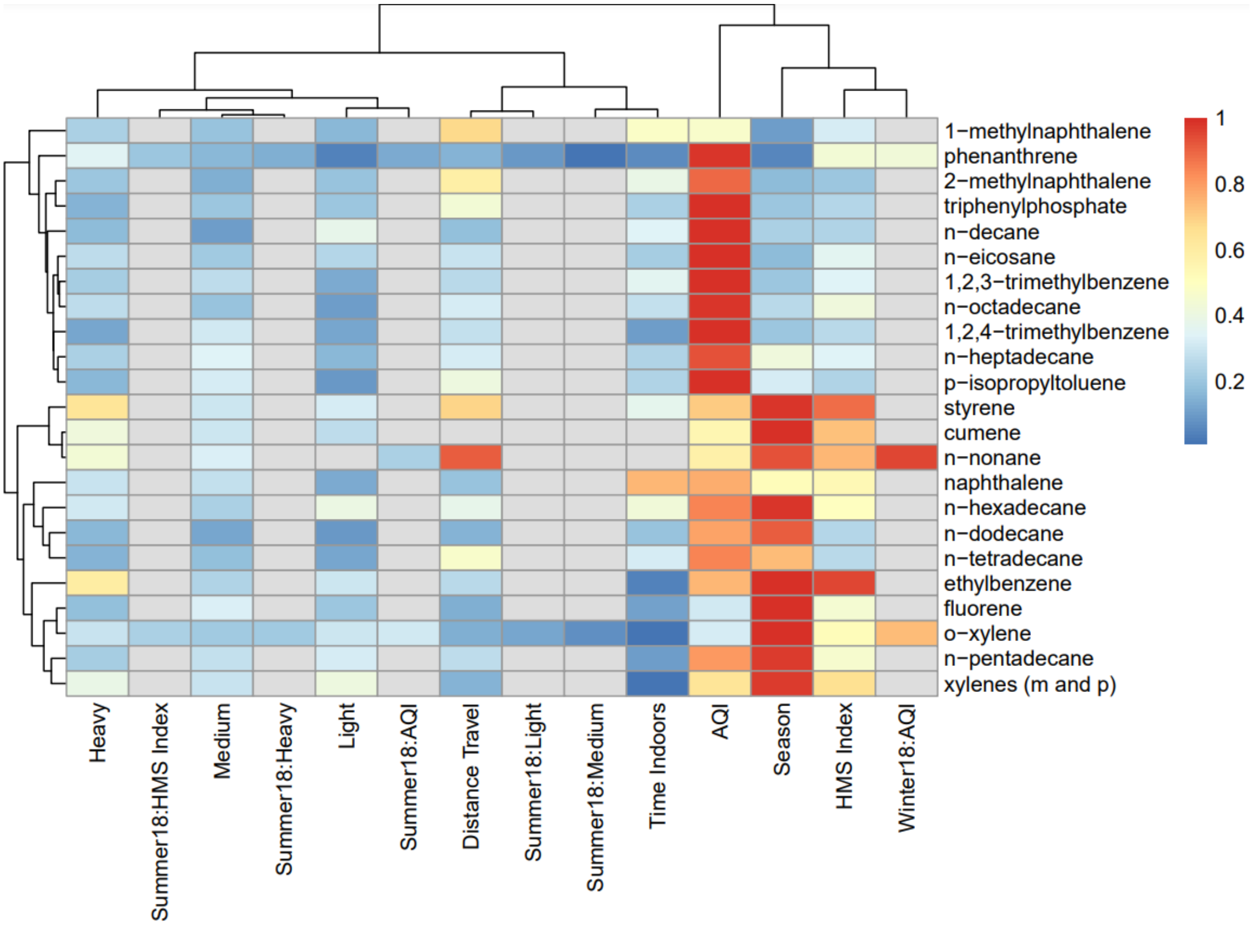

## References

Dixon HM, Scott RP, Holmes D, Calero L, Kincl LD, Waters KM, Camann DE, Calafat AM, Herbstman JB, Anderson KA (2018) Silicone wristbands compared with traditional polycyclic aromatic hydrocarbon exposure assessment methods. Anal Bioanal Chem 410:3059-3071. doi:10.1007/s00216-018-0992-z

Dixon HM, Bramer LM, Scott RP, Calero L, Holmes D, Gibson EA, Cavalier HM, Rohlman D, Miller RL, Calafat AM (2022) Evaluating predictive relationships between wristbands and urine for assessment of personal PAH exposure. Environ Int 163:107226
